# Structural and Functional Characterization of Conotoxins from *Conus achatinus* Targeting NMDAR

**DOI:** 10.3390/md18030135

**Published:** 2020-02-26

**Authors:** Xiujie Liu, Ge Yao, Kang Wang, Yanli Liu, Xiukun Wan, Hui Jiang

**Affiliations:** State Key Laboratory of NBC Protection for Civilian, Beijing 102205, China

**Keywords:** conotoxins, N-methyl-D-aspartate receptor, structure-activity relationship, *Conus achatinus*

## Abstract

Conotoxin-Ac1 and its variant conotoxin-Ac1-O6P, were isolated from the venom duct of *Conus achatinus*, a fish-hunting cone snail species collected in the Sea of Hainan, China. Conotoxin-Ac1 is linear peptide that contain 15 amino acids. In the present study, we synthesized and structurally and functionally characterized conotoxin-Ac1 as well as 19 variants. Electrophysiological results showed that conotoxin-Ac1 inhibited N-methyl-D-aspartate receptor subunit 2B (NR2B) with an IC_50_ of 8.22 ± 0.022 μM. Further structure-activity studies of conotoxin-Ac demonstrated that polar amino acid residues were important for modulating its active, and the replacement of N1, O9, E10, and S12 by Ala resulted in a significant decrease in potency to NR2B. °Furthermore, conotoxin-Ac1 and conotoxin-Ac1-O6P were tested in hot-plate and tail-flick assays to measure the potential analgesic activity to an acute thermal stimulus in a dose-dependent manner. Subsequently, the analgesic activity of conotoxin-Ac1 mutants was analyzed by the hot-plate method. The results show that N1, Y2, Y3, E10, N11, S12, and T15 play an important role in the analgesic activity of conotoxin-Ac1. N1 and S12 have significant effects on conotoxin-Ac1 in inhibiting NR2B and analgesic activity. In conclusion, we have discovered that conotoxin-Ac1 is an inhibitor of NMDAR and displays antinociceptive activity.

## 1. Introduction

Conotoxins (CTxs) are highly sophisticated set of neuropharmacological weapons formed during the long-term evolution. Most of the CTxs are composed of 12 to 40 amino acid residues and contain 2 to 3 pairs of disulfide bonds. CTxs are among smallest nucleic acid-encoded animal neurotoxin peptides, and they are also small peptides with the highest disulfide bond density [1]. CTxs can specifically act on voltage-gated ion channels (Na^+^, K^+^, Ca^2+^), ligand-gated ion channels (nAchR, 5-HT_3_R, NMDAR) and G protein-coupled receptors (neurotensin receptors and vasopressin receptors), have received extensive attention in the field of neuroscience and new drug development. For example, α, αA, and ψ-CTxs antagonize nAchR competitively and noncompetitively [2]. ω-CTxs selectively inhibit various subtypes of voltage-sensitive Ca^2+^ channels [3]. κ-CTxs selection the voltage-sensitive K^+^ channel are inhibited [4], while μ and μO-CTxs selectively act on the voltage-sensitive Na^+^ channel [5,6,7]. In addition, some Conotoxins, which do not contain or contain a pair of disulfide bonds, can selectively act on vasopressin receptors (such as conopressin) [8], NMDA receptors (such as conantokins) [9], serotonin type 3 (5-HT3) receptors [10]. CTxs can distinguish and recognize many different subtypes or heterostructures of ion channels and neural receptors and are widely used in life science research; several CTxs have been marketed or entered clinical research as analgesics and antiepileptic drugs. According to their feeding habits, cone snails are either fish-hunting (piscivorous class), mollusk-hunting (molluscivorous class), or worm-hunting (vermivorous class). Fish-hunting cone snails are not the largest category of cone snails, but the activity of CTxs on mammals is obvious. There are many types of cone snails, and each type of cone snails contains different toxins. Therefore, studying new species of cone snails means obtaining a new structure of toxins, which means that it may have new functions. *Conus achatinus* is a fish-hunting cone snails. At present, little research has been conducted at home and abroad on the toxins of this cone snail [11,12,13]. We have conducted preliminary research, taking advantage of the South China Sea resources to purify and characterize a class of containing D-amino acid CTxs. We also explored the role of these CTxs in ion channels and analgesic activity.

## 2. Results

### 2.1. Isolation, Purification and Sequencing of CTxs

#### 2.1.1. Isolation and Purification of CTxs

A 2.95 mg crude sample was obtained from the venom tube of two cone snails. The crude fraction was obtained with Superdex Peptide to obtain eight main fractions (Supplemental Figure S1). Considering the abundance of the sample, fraction #5 was taken as the research object. The collected fraction #5 was analyzed by HPLC, and the two toxins 5P1 and 5P2 were mainly contained in fraction #5 (Supplemental Figure S2). These toxins were separated and purified to obtain high-purity samples 5P1 and 5P2.

#### 2.1.2. Sequencing of CTxs 

MALDI-TOF-MS was used to measure the molecular weights of the two CTxs. After reducing the derivative with DTT and 4-vinylpyridine and measuring the molecular weight, it was found that the molecular weights of 5P1 and 5P2 did not change before and after derivatization, indicating no cysteine. For the amino acid, the molecular weight of 5P1 is 1992.11, and the molecular weight of 5P2 is 1976.11. 5P1 weighs 16 Da more than 5P2. 5P2 is a variant of 5P1 that lacks a single modification of proline hydroxylation. Next, 2 µg of each is obtained for sequencing and to determine its primary structure:

5P1: NYYLYOAROENSWWT
5P2: NYYLYPAROENSWWT

The natural 5P2 was hydrolyzed by carboxypeptidase: Since carboxypeptidase cannot hydrolyze D-type amino acids, the termination site is the D-amino residue. As shown in the results, hydrolysis was stopped when W13 was reached, and W13 was judged to be D-shaped. According to this judgment, the primary structure of 5P2 should be NYYLYPAROENSWWT, where W is a D-amino acid, O is hydroxyproline. Chemically synthesized 5P2, identified by HPLC, was able to coelute with natural toxin 5P2, indicating a consistent structure. The same strategy was used to synthesize 5P1: NYYLYOAROENSWWT, which was identified by HPLC as coeluting with the natural toxin 5P1, indicating a consistent structure (Supplemental Figures S3 and S4). 5P1 and 5P2 are mature peptides, isolated from Conus achatinus.Using NCBI website, multimode search was conducted, the full-length precursor sequence of 5P1 and 5P2 was found. Wu et al [14] reported that Ac3.1 was identified by cDNA, and the deduced amino acid sequences of the cloned conotoxins Ac3.1: LGVLVTIFLVLFPMATLQLDGDQTADRHAGERDQDPLEQYRNLKHVLRRTRNYYLYPARPENSWWT. Based on Ac3.1, we named 5P1 as conotoxin-Ac1, and 5P2 is the variant of 5P1, named 5P2 as conotoxin-Ac1-O6P.

### 2.2. Chemical Synthesis of Conotoxin-Ac1, Its Variant and Its Mutants

According to the sequence of each peptide in Table 1, peptides were synthesized by solid-phase synthesis. The purity of each peptide was greater than 95% after HPLC analysis and determined by mass spectrometry (Supplemental MS data). The overall conformation of 20 CTxs was examined by CD spectroscopy (Table 2). The secondary structure of conotoxin-Ac1 was 44.9% α-helix, 27.7% β-sheet, 15% β-turn and 12.4% random coil. Interestingly, the secondary structure of conotoxin-Ac1-Y5A was 46.8% β-sheet, 27.3% β-turn, 17.8% random coil, and 8.1% α-helix. The percentages of β-sheets and β-turns in conotoxin-Ac1-Y5A are higher than those in conotoxin-Ac1, and the percentage of α-helices in conotoxin-Ac1-Y5A is considerably lower than that in conotoxin-Ac1. The secondary structures of 19 CTxs—except for conotoxin-Ac1-Y5A—are mainly α-helices and β-sheets. Only 41.2% of the α-helices of conotoxin-Ac1-O9A and 37.1% of the α-helices of conotoxin-Ac1-E10A are lower than 44.9% of the α-helices of conotoxin-Ac1.

### 2.3. Electrophysiology Experiment

#### 2.3.1. Conotoxin-Ac1 and Conotoxin-Ac1-O6P act on NMDAR Ion Channels

The hippocampus of newborn SD rats was isolated, and hippocampal neuron cells were obtained. After incubation, the effect of 10 μM conotoxin-Ac1 and conotoxin-Ac1-O6P on the activity of Na^+^, K^+^, Ca^2+^, and NMDAR ion channels was analyzed using whole-cell patch-clamp technology (Table 3). The experimental results show that conotoxin-Ac1 and conotoxin-Ac1-O6P have almost no inhibitory effect on Na^+^ and K^+^, conotoxin-Ac1-O6P has a relatively low inhibitory effect on Ca^2+^ ion channels, conotoxin-Ac1 and conotoxin-Ac1-O6P have the most obvious inhibitory activity on NMDAR ion channels, and 10 μM conotoxin-Ac1 inhibited the current of NMDAR ion channels by 56.90%. Therefore, we believe that conotoxin-Ac1 and conotoxin-Ac1-O6P act on the NMDAR ion channel.

#### 2.3.2. Conotoxin-Ac1 and Conotoxin-Ac1-O6P Act on the NR2A and NR2B Subtype

HEK293 cells stably expressing NR2B/NR2A were used, and the effect of 10 μM conotoxin-Ac1 on the activity of NR2A and NR2B ion channels was analyzed by the whole-cell patch clamp technique (Table 3). The experimental results show that 10 μM conotoxin-Ac1 has a higher activity inhibition rate of NR2B ion channels of up to 51.69%, while a lower activity inhibition rate of NR2A ion channels, that is, only 7.22%. Conotoxin-Ac1 has a high inhibition rate to NR2B than NR2A. The inhibition rate of NR2A and NR2B ion channels was lower in 10 μM conotoxin-Ac1-O6P. The difference between conotoxin-Ac1 and conotoxin-Ac1-O6P is that only the amino acid residue at position 6 in conotoxin-Ac1 is hydroxyproline, the amino acid residue at position 6 in conotoxin-Ac1 is proline, and the percentage of inhibition of the NR2B ion channel activity between 10 μM conotoxin-Ac1 and 10 μM conotoxin-Ac1-O6P is different by almost five-fold. The experimental results show that the hydroxyproline residue at position 6 in conotoxin-Ac1 is its key active amino acid residue, which plays an important role in inhibiting NR2B activity.

#### 2.3.3. Effect of Conotoxin-Ac1 Mutants on NR2B

**Figure 1:**
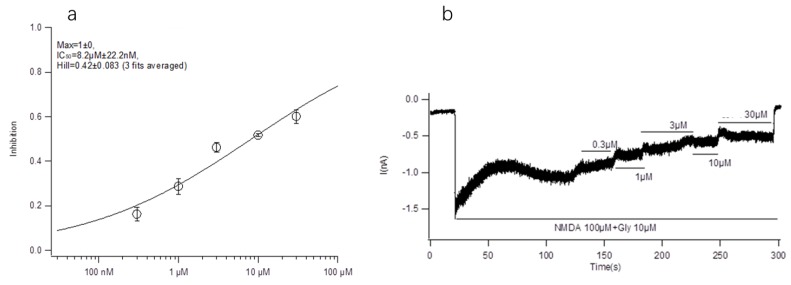
Effect of conotoxin-Ac1 on NR2B in HEK293 cells by Electrophysiology experiment. (**a**) The dose-response relationship of the effects of conotoxin-Ac1 on NR2B. (**b**) Electrophysiological profile of conotoxin-Ac1 on NR2B.

HEK293 cells stably expressing NR2B/NR2A were used, and the activity inhibition rate of conotoxin-Ac1 on NR2B ion channels was analyzed by the whole-cell patch clamp technique. The experimental results are shown in the figure. The IC_50_ value of conotoxin-Ac1 on the activity of NR2B ion channels is 8.22 ± 0.022 μM (Figure 1). In this work, the patch clamp technique was used to detect the influence of conotoxin-Ac1, its variant, and its mutants on NR2B in HEK293 cells. The results showed that conotoxin-Ac1, its variant, and its mutants could induce NR2B channel closure. We also analyzed the affinities of conotoxin-Ac1 mutants with human NR2B (Table 4). Compared with conotoxin-Ac1, the affinity between human NR2B and conotoxin-Ac1 mutants was significantly reduced. Although the affinity between the human NR2B and conotoxin-Ac1 mutants was weaker, the affinity of most mutants and conotoxin-Ac1 was on the same order of magnitude. The inhibition of conotoxin-Ac1-S12A was 25 times weaker than that of conotoxin-Ac1, and the affinity of NR2B and conotoxin-Ac1-N1A (3) or conotoxin-Ac1-O9A (10) or conotoxin-Ac1-E10A (11) was almost 10 times weaker than that of conotoxin-Ac1. The inhibition of conotoxin-Ac1-15* (17), conotoxin-Ac1-E10γ (18), conotoxin-Ac1-E10γW14γ (19) and conotoxin-Ac1-E10γW14γ15* (20) was clearly weaker than that of conotoxin-Ac1.

### 2.4. Animal Experiment

#### 2.4.1. Overall Animal Activity of Conotoxin-Ac1 and Conotoxin-Ac1-O6P

Both CTxs, conotoxin-Ac1 and conotoxin-Ac1-O6P, showed signs of mental weakness and a sluggish response after lateral brain administration. The time of disappearance of the righting reflex in mice was time (Table 5). The experimental results showed that recorded as sleep latency, and the duration of disappearance of the righting reflex was recorded as sleep after the lateral brain injection of conotoxin-Ac1 and conotoxin-Ac1-O6P, the sleep latency of the mice was significantly shortened, while the sleep time was longer than that of the control group, but the difference was statistically significant. The sleep time of the two groups of low-dose mice was longer than 10 min, while the sleep time of the conotoxin-Ac1 high-dose group was 36.87 min, which was longer than the 21.54 min of the conotoxin-Ac1 high-dose group, and the difference was significant, suggesting that conotoxin-Ac1 has higher overall animal activity than conotoxin-Ac1-O6P.

#### 2.4.2. Animal Analgesic Activity

##### Analgesic Activity of Conotoxin-Ac1 and Conotoxin-Ac1-O6P Detected by the Hot-plate Method

**Figure 2:**
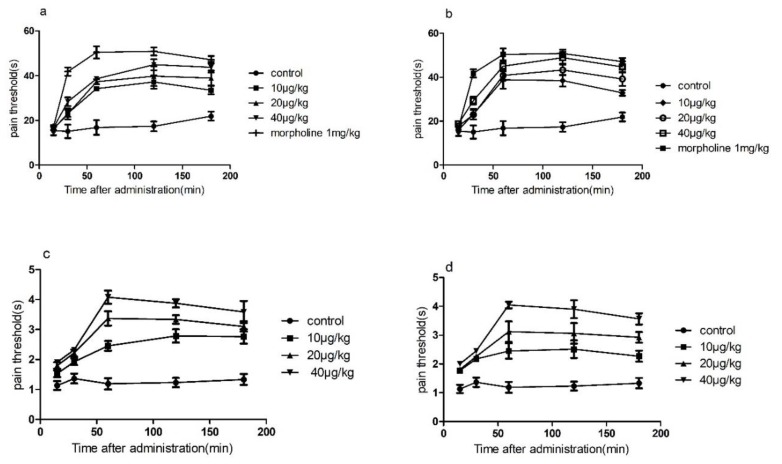
The time course of the dose-dependent analgesic effect of conotoxin-Ac1 and conotoxin-Ac1-O6P in mice. (**a**) The dose-dependent analgesic effect of conotoxin-Ac1 by hot-plate method. (**b**) The dose-dependent analgesia effect of conotoxin-Ac1-O6P by hot-plate method. (**c**) The dose-dependent analgesic effect of conotoxin-Ac1 by tail-flick method. (**d**) The dose-dependent analgesia effect of conotoxin-Ac1-O6P by tail-flick method.

Before the experiment, female mice with a normal pain threshold <30 s were preselected and randomly divided into 8 groups of 6 mice in each group. Each group was administered by lateral injection (Figure 2a,b and Supplemental Table S1). The pain threshold was 15, 30, 60, 120, and 180 min after the drug, and the t-test was performed with the normal saline group. As shown in Figure 2, lateral injection of conotoxin-Ac1 and conotoxin-Ac1-O6P at 10, 20, and 40 μg/kg doses after 15, 30, 60, 120, and 180 min significantly increased the pain threshold, and there were significant differences compared with normal saline. The pain thresholds were all lower than the positive control, 1 mg/kg morphine.

##### Analgesic Activity of Conotoxin-Ac1 and Conotoxin-Ac1-O6P Detected by the Tail-flick Method

Before the experiment, female mice with a normal pain threshold <1 s were preselected and randomly divided into 7 groups of 6 mice each. The tail-flick time of the mice was recorded at 15, 30, 60, 120, and 180 min after administration (Figure 2c,d and Supplemental Table S2). Pain thresholds were calculated at 15, 30, 60, 120, and 180 min after administration, and t-tests were performed with the saline group. As shown in Figure 2, lateral injection of conotoxin-Ac1 and conotoxin-Ac1-O6P at 10, 20, and 40 μg/kg doses after 15, 30, 60, 120, and 180 min significantly increased the pain threshold, and there were significant differences compared with the normal saline group. However, the pain threshold of each group was lower than the positive control, 1 mg/kg morphine.

##### Analgesic Activity of Conotoxin-Ac1 Mutants Detected by the Hot-plate Method

Time-analgesic activity showed that the conotoxin-Ac1 mutants had an analgesic effect after 30 min of administration, and the analgesic effect was best after 1–2 h of administration. Each conotoxin-Ac1 mutants had a sustained analgesic effect at a dose of 10 μg/kg for more than 3 h (Table 6). The conotoxin-Ac1 mutants modified according to the alanine replacement method have analgesic effects less than conotoxin-Ac1, and only conotoxin-Ac1-E10A (11) and conotoxin-Ac1-T15A (16) after 60 min of administration, the pain threshold is lower than conotoxin-Ac1, and there is a significant difference (*p* < 0.05). In addition, the analgesic effects of conotoxin-Ac1-15* (17), conotoxin-Ac1-E10γ (18), conotoxin-Ac1-E10γW14γ (19), and conotoxin-Ac1-E10γW14γ15* (20) are better than conotoxin-Ac1, and conotoxin-Ac1-E10γW14γ (19) and conotoxin-Ac1-E10γW14γ15* (20) are administered for 30 min. The pain thresholds after 30, 60, 120, and 180 min were significantly higher than the pain thresholds of conotoxin-Ac1 (*p* < 0.05 or *p* < 0.01). conotoxin-Ac1-E10γW14γ15* (20) was administered at 60 and 120 min. The pain threshold after 180 min was better than that of the positive control (morphine).

## 3. Discussion

Using NCBI website, multimode search was conducted, conotoxin-Ac1 was identified as the mature region of the reported sequence of conotoxin Ac3.1 from the same species *Conus achatinus* (Sequence ID: P0CH24.1) [14]. The full-length cDNA of Ac3.1 was cloned from the venom-duct transcriptome with a combined PCR and RACE approach, and the amino acid sequence of Ac3.1 precursor was deduced and assigned to M-superfamily in the previous research. In this study, we performed peptides purification and sequencing to analyze and identify the mature toxins conotoxin-Ac1 and conotoxin-Ac1-O6P, located the accurate cleavage site of mature peptide formation, but also confirmed the high expression of conotoxin Ac3.1 preliminarily identified in the transcript level. It is reported in the literature that most CTxs contain disulfide bonds [15], while conotoxin-Ac1 in *Conus achatinus* is cysteine-free linear toxin. Linear toxins, as in *Conus achatinus*, are presented in high abundance. This situation is still rare and has strong research value. conotoxin-Ac1 contains 15 amino acid residues, including six aromatic amino acid residues, N-terminal (YYLY) and C-terminal (WW) are highly hydrophobic and lipophilic, while the intermediate (ROENS) is hydrophilic. Current research indicates that CTxs do not contain cysteine in cone snails can be roughly divided into six categories (Table 7). The activity of these CTxs are also different. Mo1659, which acts on the K^+^ channel; Conomap-Vt can excite muscle tissue, but its role in receptors has not been determined; contulakin-G acts on the neurotensin receptor [16]; conantokins can block NMDAR. According to current literatures, conantokins are the only peptide-type NMDAR inhibitors with strong neuroprotective effects, and also have good anti-pain effects [17]. By electrophysiological experiments, we found that conotoxin-Ac1 can act on NMDAR ion channel. in addition to conantokins, which are reported in the literature, conotoxin-Ac1 is natural peptide that inhibits NMDAR activity.

Firstly, design and chemical synthesis of conotoxin-Ac1 mutants was performed. The alanine replacement method replaces each non-alanine residue site with alanine, removes the active group on the side chain, and replaces it with a small methyl group without other functional groups. This methyl group is small and can be used to identify the positions of key amino acids. We used the alanine substitution method to mutate all non-alanine residues in conotoxin-Ac1 through solid-phase synthesis of peptides, numbered 3–16. Prior results indicate that conantokins are antagonists of NMDAR, in which the C-terminal residues of this class of peptides are amidated, also, the conotoxin-Ac1 C-terminal Thr15 is mutated to amidated Thr15, which is conotoxin-Ac1-15* (17). Conantokins all contain γ-carboxyglutamic acid, which is a site for binding to metal ions. It was found that the γ-carboxyglutamic acid arrangement must meet the requirements of “i, i + 4, i + 7, i + 11”; therefore, Glu10 is mutated to γ-carboxyglutamic acid, or both Glu10 and Trp14 are mutated to γ-carboxyglutamic acid, which are conotoxin-Ac1-E10γ (18), conotoxin-Ac1-E10γW14γ (19), or conotoxin-Ac1-E10γW14γ15 * (20). The accuracy of each synthesized CTx polypeptide was analyzed by HPLC and MS, and then the changes in the secondary structure of conotoxin-Ac1 by each amino acid were analyzed by CD spectroscopy. In the PB buffer solution, the proportion of the α-helix structure in conotoxin-Ac1 was 44.9%. Except for conotoxin-Ac1-Y5A (7), the other mutants also had α-helices as their main secondary structure. Although conotoxin-Ac1-Y2A (4), conotoxin-Ac1-Y3A (5) and conotoxin-Ac1-Y5A (7) all mutate tyrosine to alanine, their effects on the secondary structure are not the same, suggesting that amino acids have a secondary effect. The influence of structure is related not only to the type of amino acid but also to the site where it is located.

The main purpose of this paper is to explore the structure-activity relationship between conotoxin-Ac1 and its target. We first analyzed the effects of 10 μM conotoxin-Ac1 and conotoxin-Ac1-O6P on the activity of Na^+^, K^+^, Ca^2+^, and NMDAR ion channels using whole-cell patch-clamp technology. The results showed that 10 μM conotoxin-Ac1 and conotoxin-Ac1-O6P had almost no inhibitory effect on Na^+^ and K^+^, 10 μM conotoxin-Ac1-O6P had an inhibitory effect on Ca^2+^ ion channels, and 10 μM conotoxin-Ac1 had a current inhibition percentage of 56.90% on NMDAR ion channels. Therefore, the NMDAR ion channel is the active channel of conotoxin-Ac1. Subsequently, the effects of 10 μM conotoxin-Ac1 and conotoxin-Ac1-O6P on NMDAR ion channel subtypes NR2A and NR2B were investigated. The results showed that the activity inhibition rate of the NR2B ion channel was increased by 10 μM conotoxin-Ac1 to 51.69%, while that of the NR2A ion channel was only 7.22%, the activity inhibition rate of the NR2B ion channel was increased by 10 μM conotoxin-Ac1-O6P to 10.63%, while that of the NR2A ion channel was only 1.85%, The result showed that the absence of the hydroxyl group on O6 of conotoxin-Ac1 causes a five-fold difference at the NMDAR subtypes NR2A and NR2B. The IC50 value of conotoxin-Ac1 on the activity of the NR2B ion channel was 8.22 ± 0.022 μM. It is suggested that conotoxin-Ac1 acts on the NR2B subtype. Finally, we investigated the effect of conotoxin-Ac1 mutants on NR2B and discussed the structure-activity relationship between conotoxin-Ac1 and NR2B. Among the alanine substitution mutants numbered 3–16, conotoxin-Ac1-N1A (3), conotoxin-Ac1-O9A (10), conotoxin-Ac1-E10A (11) and conotoxin-Ac1-S12A (13) all inhibited NR2B. Compared with the inhibitory rate of conotoxin-Ac1 on NR2B, the ratio is reduced by 10 times, and the numbers 3, 10, 11, and 13 are all mutated from polar amino acids to nonpolar amino acids, suggesting that polar amino acids have an important effect on the activity of conotoxin-Ac1. Although all mutations were o to A, the inhibition rate of conotoxin-Ac1-O6A (8) on NR2B was 18.98% greater than that of conotoxin-Ac1-O9A (10) on NR2B, which was approximately 4.27%—approximately four times greater. The abovementioned experimental results show that N1, O9, E10, and S12 are the key active amino acids of conotoxin-Ac1, which play an important role in inhibiting the activity of NR2B. In addition, our mutants, numbered 17–20 based on the structural rule of the NMDAR antagonist conantokins did not show strong anti-NR2B activity. The experimental results suggest that conotoxin-Ac1 and conantokins are not the same NMDAR antagonist.

The results of conotoxin-Ac1 and conotoxin-Ac1-O6P animal overall activity evaluation showed that conotoxin-Ac1 and conotoxin-Ac1-O6P can significantly shorten sleep latency and prolong sleep time. It is shown that conotoxin-Ac1 and conotoxin-Ac1-O6P have stabilization effects. The analgesic activity of conotoxin-Ac1 and conotoxin-Ac1-O6P was examined by the hot-plate method and tail-burn method. The results showed that 10, 20, and 40 μg/kg doses of conotoxin-Ac1 and conotoxin-Ac1-O6P all had analgesic activity, and there were significant differences compared with the control group. Electrophysiological results showed that conotoxin-Ac1-O6P, and the absence of the hydroxyl group on O6 of conotoxin-Ac1, caused a five-fold difference at the NMDAR, but the analgesic activity of these two conotoxins are very similar, Therefore, we believe that the relationship between conotoxin-Ac1 analgesic activity and conotoxin-Ac1 inhibitory activity of NMDAR still needs to be studied in subsequent experiments. Subsequently, the analgesic activity of 10 μg/kg doses of the conotoxin-Ac1 mutants was analyzed by the hot-plate method. The conotoxin-Ac1 mutants numbered 3–16 were less than conotoxin-Ac1. conotoxin-Ac1-N1A (3), conotoxin-Ac1-Y2A (4), conotoxin-Ac1-Y3A (5), conotoxin-Ac1-N11A (12), and conotoxin-Ac1-S12A (13) not only reduce analgesic activity but also reach the maximum analgesic effect after 60 min of administration, and conotoxin-Ac1 reaches the maximum analgesia after 120 min of administration. The effects are notably different. In addition, the pain threshold of conotoxin-Ac1-E10A (11) and conotoxin-Ac1-T15A (16) was lower than that of conotoxin-Ac1 after 60 min of administration, and there were significant differences (*p* < 0.05). The above results show that N1, Y2, Y3, E10, N11, S12, and T15 play important roles in the analgesic activity of conotoxin-Ac1. Combined with electrophysiological experiments, N1 and S12 have significant effects on conotoxin-Ac1 in inhibiting NR2B and analgesic activity, specifically conotoxin-Ac1-15* (17), conotoxin-Ac1-E10γ (18), conotoxin-Ac1-E10γW14γ (19), and conotoxin-Ac1-E10γW14γ15* (20) Although these compounds have strong NR2B-inhibitory activity, they have analgesic activity, but it was higher than that of conotoxin-Ac1; furthermore, the pain thresholds of conotoxin-Ac1-E10γW14γ(19), conotoxin-Ac1-E10γW14γ15* (20) after administration for 30, 60, 120, and 180 min were significantly higher than the pain threshold of 5P1 (*p* < 0.05). It is suggested that c-terminal amidation and γ amino acid have a strong effect on the analgesic activity of conotoxin-Ac1.

## 4. Materials and Methods 

### 4.1. Isolation, Purification and Sequencing of CTxs of Conus Achatinus

#### 4.1.1. Isolation and Purification of CTxs

Conus achatinus is collected from the sea area near Qionghai, Hainan, with a body length of 6.5 cm, a shoulder height of 5.5 cm, and a maximum diameter of 3.5 cm at the center. The dissected venom tube is 7 cm in length, 0.5 cm in diameter, and 1.5 cm in length. The tube is cut into 2 mm lengths and placed in a 2 mL EP tube. One milliliter of 35% acetonitrile (containing 0.1% TFA) was added. Then, the samples were centrifuged at 13,000× *g* at 4 °C for 15 min, and the supernatant was collected and freeze-dried. The lyophilized sample was dissolved in 300 µL of 30% acetonitrile (containing 0.1% TFA) and centrifuged at 12,000× *g* for 5 min, and the supernatant was separated using a Superdex Peptide^®^ 7.5 × 300 mm column. The fractions were collected and labeled as #1, #2, #3, etc,, concentrated under reduced pressure and freeze-dried for later use. The #5 fraction was further subjected to gradient separation with an analytical column to obtain 5P1 and 5P2.

#### 4.1.2. Sequencing of CTxs

MALDI-TOF-MS was used to detect the molecular weights of 5P1 and 5P2. The obtained 5P1 and 5P2 were reduced by DTT under alkaline conditions (pH 8.0), derivatized with 4-vinylpyridine, and then sequenced by an ABI491 protein sequencer using a standard Edman degradation sequencing method. Subsequently, a single toxin was digested with carboxypeptidase, 5 µg of toxin peptide was dissolved in 10 µL of 0.05 M citrate buffer (pH 6.0), and then 10 µL of citrate buffer containing 0.5 units of carboxypeptidase Y was added. The reaction was carried out at a temperature of 0.2 °C, and 0.2 µL of the reaction solution was taken every few minutes. The reaction was terminated with CCA, and the molecular weight was detected by mass spectrometry.

### 4.2. Chemical Synthesis of Conotoxin-Ac1, Its Variant and Its Mutants

The SPPS method was used, and according to the Fmoc-TFA strategy, the synthesis method and process were performed with reference to the literature [27]. First, the alanine substitution method was used to artificially synthesize all non-alanine residues in conotoxin-Ac1, numbered 3–16. Second, according to the existing literature, the residues were mutated to the corresponding amino acids, numbered 17–20. For specific numbers and corresponding sequences, see Table 2. The synthetic peptides were verified by HPLC and MS, and the changes in the secondary structure of the peptides were analyzed by CD spectroscopy.

### 4.3. Electrophysiology Experiment

#### 4.3.1. Discovery of Conotoxin-Ac1 and Conotoxin-Ac1-O6P Acting on Ion Channels

Newborn SD rat hippocampal cells were isolated for primary culture of hippocampal neurons. Isolation and culture methods were performed with reference to the literature [28]. The whole-cell patch-clamp uses an Axon700B amplifier, and the data processing software is Clampfit10.2 and Sigmaplot10.0. The glass electrode is drawn in four steps using a shutter P-97 electrode drawing instrument and thermally polished by MF-900 to facilitate sealing. conotoxin-Ac1 was dissolved in extracellular fluid at a concentration of 10 μM for subsequent detection. The specific experimental operation of the patch clamp was performed in reference [29].

##### Recording Solution of Hippocampal Neuron Whole Cell

Intracellular and extracellular solution required for recording Nav current. External solution: 140 mM NaCl, 3.5 mM KCl, 1 mM MgCl_2_, 2 mM CaCl_2_, 10 mM Glucose, 10 mM HEPES, 1.25 mM NaH_2_PO_4_, pH = 7.4 with NaOH. intracellular solution: 50 mM CsCl, 10 mM NaCl, 10 mM HEPES, 60 mM CsF, 20 mM EGTA, pH = 7.2 with CsOH.

Intracellular and extracellular solution required for recording Cav current. External solution: 140 mM TEA-Cl, 2 mM MgCl_2_, 10 mM CaCl_2_, 10 mM HEPES,5 mM Glucose, pH = 7.4 with TEA-OH.intracellular solution: 110 mM CsCl, 1 mM MgCl_2_, 10 mM HEPES, 4 mM Na_2_-ATP, 10 mM EGTA, 0.3 mM Na_2_-GTP, 5 mM Phosphoceatine disodium salt, 7 mM Creatine, pH = 7.2 with CsOH.

Intracellular and extracellular solution required for recording Kv current. External solution: 5 mM NaCl, 140 mM K-Gluconate, 1 mM MgCl_2_, 0.1 mM CaCl_2_, 10 mM EGTA,10 mM HEPES, 2 mM Mg-ATP, 300nM TTX, pH = 7.4 with NaOH. intracellular solution: 20 mM KCl, 115 mM K-aspartic, 10 mM HEPES, 1 mM MgCl_2_, 5 mM EGTA, 2 mM Na_2_-ATP, pH = 7.2 with KOH.

Intracellular and extracellular solution required for recording NMDAR current. External solution: 140 mM NaCl, 4 mM KCl, 2 mM CaCl_2_, 5 mM Glucose, 10 mM HEPES, pH = 7.4 with NaOH. Intracellular solution: 110 mM CsMes, 10 mM NaCl, 2 mM MgCl_2_, 10 mM HEPES, 10 mM EGTA, 2 mM Na_2_-ATP, 0.2 mM Na_2_-GTP, pH = 7.2 with CsOH.

#### 4.3.2. Screening for Conotoxin-Ac1 Acting NMDAR Subtypes

For the expression of NR2B/NR2A in HEK293 cells, the linearized plasmids were transcribed using the Lipofectamine 2000 transcription kit, as previously described [30]. Cells were separated with TrypLE Express before patch clamp detection. A total of 3 × 10^3^ cells was spread on coverslips and cultured in 24-well plates. After 18 h, experimental detection was performed. conotoxin-Ac1 was dissolved in extracellular fluid to a concentration of 10 μM. The experiment used voltage clamp mode, the cell membrane potential was clamped at −70 mV, and the recording was performed in Gap free mode for a duration of 150–300 s. Immediately after the voltage was maintained for 10–20 s, 100 μM NMDA and 10 μM Gly were administered followed by stimulation with 100 μM NMDA and 10 μM Gly and 10 μM conotoxin-Ac1.

#### 4.3.3. Effect of Conotoxin-Ac1 Mutants on NR2B

HEK293 cells expressing NR2B were selected, and 19 peptide samples of conotoxin-Ac1 mutants were lysed with extracellular fluid. The voltage clamp mode was used in the experiment, and the cell membrane potential was clamped at −70 mV and recorded in Gap free mode, duration a of 150 to 300 s. Immediately after the voltage was maintained for 10–20 s, 100 μM NMDA and 10 μM Gly were administered followed by 100 μM NMDA and 10 μM Gly and 0.3, 1, 3, 10, and 30 μM conotoxin-Ac1 for stimulation. The remaining 19 peptide samples were formulated as 100 μM NMDA and 10 μM Gly and 10 μM test peptide samples for stimulation.

### 4.4. Animal Experiment

#### 4.4.1. Animal Overall Activity

The method of cranial administration was used to observe the behavior of animals to evaluate the toxins. conotoxin-Ac1 and conotoxin-Ac1-O6P were dissolved in physiological saline. Thirty Kunming mice of similar size, 18 ± 2 g, male and female. All animal studies were in accordance with the NIH guide for the care and use of laboratory animals and were approved by the Beijing Institute of Technology Animal Institute Committee (SYXK-BIT-20161008001). Mice were selected and randomly divided into 5 groups, namely, the control group, conotoxin-Ac1 at 250 and 500 µg/kg doses, and conotoxin-Ac1-O6P at 250 and 500 µg/kg doses. A graduated needle was used to inject the corresponding volume into the lateral ventricle, and the control group was injected with the same dose of normal saline. After the injection was completed, the mice were returned to their cages, and their behaviors and symptoms were observed and recorded. If the mouse is not supine within 1 min, it means that the righting reflex has disappeared. Time was recorded as the sleep latency; the duration of the disappearance of the righting reflex was recorded as the sleep time, and the sleep latency and sleep time of each group falling asleep were recorded separately.

#### 4.4.2. Animal Analgesic Activity

##### Analgesic Activity of Conotoxin-Ac1 and Conotoxin-Ac1-O6P Detected by the Hot-Plate Method

The analgesic activity of conotoxin-Ac1 and conotoxin-Ac1-O6P was evaluated with the classical mouse hot-plate method [31]. The pain threshold, the latency at which the mouse licked its hind paw or jumped, was determined on a 55 °C hot plate. Mice with a threshold <30 s were divided into eight groups, and each group contained six mice. By intracranial administration, mice in group 1 were given saline as a control, while mice in group 2 were given morphine at a 10 μg/kg dose as a positive control. Groups 3, 4, and 5 were given conotoxin-Ac1 at 10, 20, and 40 μg/kg doses, and groups 6, 7, and 8 were given conotoxin-Ac1-O6P at 10, 20, and 40 μg/kg doses. The response time (RT) was determined at 15, 30, 60, 120, and 180 min after administration. Data are expressed as means ± SD, and the t-test was used to determine statistical significance.

##### Analgesic Activity of Conotoxin-Ac1 and Conotoxin-Ac1-O6P Detected by the Tail-Flick Method

The analgesic activity of conotoxin-Ac1 and conotoxin-Ac1-O6P was evaluated with the classical mouse tail-flick method [32]. The pain threshold, the latency at which the mouse licked its hind paw or jumped, was determined on a 55 °C hot plate. The rats were placed in a fixed cylinder, the tail tips of the mice were exposed, and 3 cm of the tail tips of the mice were placed into a preheated water bath at 55 °C. Mice with a threshold <2 s were divided into seven groups, and each group contained six mice. By intracranial administration, mice in group 1 were given saline as a control, while mice in group 2 were given morphine at a 1 mg/kg dose as a positive control. Groups 3, 4, and 5 were given conotoxin-Ac1 at 10, 20, and 40 μg/kg doses, and groups 6, 7, and 8 were given conotoxin-Ac1-O6P at 10, 20, and 40 μg/kg doses. The tail-flick response time was determined at 15, 30, 60, 120, and 180 min after administration. Data are expressed as means ± SD, and the t-test was used to determine significance.

##### Analgesic Activity of Conotoxin-Ac1 Mutants Detected by the Hot-Plate Method

The analgesic activity of conotoxin-Ac1 mutants was evaluated with the classical mouse hot-plate method [31]. The pain threshold, the latency at which the mouse licked its hind paw or jumped, was determined on a 55 °C hot plate. Mice with a threshold <30 s were divided into eight groups, and each group contained six mice. By intracranial administration, mice in each group were given each mutant at a 10 μg/kg dose. The response time (RT) was determined at 15, 30, 60, 120, and 180 min after administration. Data are expressed as means ± SD, and the t-test was used to determine statistical significance.

### 4.5. Statistical Analysis

The results are expressed as means ± standard deviation. The significance values were calculated using SPSS 17.0 software. Data were analyzed by homogeneity of variance and one-way analysis of variance (ANOVA). P-values less than 0.05 were considered to be significant.

## Figures and Tables

**Table 1:** The amino acid sequences of conotoxin-Ac1, its variant and its mutants.

No.	Name	Molecular Weight	Sequence
1	conotoxin-Ac1	1992.11	NYYLYOAROENSWWT
2	conotoxin-Ac1-O6P	1976.11	NYYLYPAROENSWWT
3	conotoxin-Ac1-N1A	1758.84	AYYLYOAROENSWWT
4	conotoxin-Ac1-Y2A	1705.77	NAYLYOAROENSWWT
5	conotoxin-Ac1-Y3A	1705.77	NYALYOAROENSWWT
6	conotoxin-Ac1-L4A	1755.79	NYYAYOAROENSWWT
7	conotoxin-Ac1-Y5A	1705.77	NYYLAOAROENSWWT
8	conotoxin-Ac1-O6A	1852.95	NYYLYAAROENSWWT
9	conotoxin-Ac1-R8A	1712.76	NYYLYOAAOENSWWT
10	conotoxin-Ac1-O9A	1852.95	NYYLYOARAENSWWT
11	conotoxin-Ac1-E10A	1739.83	NYYLYOAROANSWWT
12	conotoxin-Ac1-N11A	1754.84	NYYLYOAROEASWWT
13	conotoxin-Ac1-S12A	1781.87	NYYLYOAROENAWWT
14	conotoxin-Ac1-W13A	1682.73	NYYLYOAROENSAWT
15	conotoxin-Ac1-W14A	1682.73	NYYLYOAROENSWAT
16	conotoxin-Ac1-T15A	1767.84	NYYLYOAROENSWWA
17	conotoxin-Ac1-15 *	1796.88	NYYLYOAROENSWWT *
18	conotoxin-Ac1-E10γ	1797.87	NYYLYOAROγNSWWT
19	conotoxin-Ac1-E10γW14γ	1740.77	NYYLYOAROγNSWγT
20	conotoxin-Ac1- E10γW14γ15 *	1739.77	NYYLYOAROγNSWγT *

* C-terminal amidation; γ: γ-carboxyglutamic acid; W: D-type tryptophan; O: hydroxyproline.

**Table 2:** CD spectrum analysis of conotoxin-Ac1, its variant and its mutants.

No.	Name	α-helix (%)	β-sheet (%)	β-turn (%)	Random Coil (%)
1	conotoxin-Ac1	44.9	27.7	15	12.4
2	conotoxin-Ac1-O6P	51	21.9	16.1	11
3	conotoxin-Ac1-N1A	54.7	35	8.1	2.2
4	conotoxin-Ac1-Y2A	48.4	23.4	16.3	11.9
5	conotoxin-Ac1-Y3A	48.9	23	16.6	11.5
6	conotoxin-Ac1-L4A	49.6	25.7	13.2	11.5
7	conotoxin-Ac1-Y5A	8.1	46.8	27.3	17.8
8	conotoxin-Ac1-O6A	46.6	26.6	6.4	20.4
9	conotoxin-Ac1-R8A	54.4	23.5	14.1	8
10	conotoxin-Ac1-O9A	41.2	27.4	16.2	15.2
11	conotoxin-Ac1-E10A	37.1	30.2	16.1	16.6
12	conotoxin-Ac1-N11A	51.1	24.6	14.3	10
13	conotoxin-Ac1-S12A	46.7	25.9	16.8	10.6
14	conotoxin-Ac1-W13A	49.2	16.2	23.1	11.5
15	conotoxin-Ac1-W14A	45.9	23.1	16.6	14.4
16	conotoxin-Ac1-T15A	49	21.3	13.6	16.1
17	conotoxin-Ac1-15 *	48.7	26.7	12.7	11.9
18	conotoxin-Ac1-E10γ	50.7	23.1	9.4	16.8
19	conotoxin-Ac1-E10γW14γ	52.9	26.8	9.9	10.4
20	conotoxin-Ac1-E10γW14γ15 *	47.2	18.2	18.1	16.5

* C-terminal amidation; γ: γ-carboxyglutamic acid; O: hydroxyproline.

**Table 3:** The electrophysiological result of 5P1 and conotoxin-Ac1-O6P inhibiting ion channels.

Groups	The Inhibition of Currents (%)					
	Nav	Kv	Cav	NMDAR	NR2A	NR2B
10 μM conotoxin-Ac1	0.55	5.50	6.70	56.90	7.22	51.69
10 μM conotoxin-Ac1-O6P	2.69	5.71	17.89	14.37	1.85	10.63

**Table 4:** The electrophysiological result of conotoxin-Ac1, its variant and its mutants inhibiting NR2B (Mean ± SD).

No.	Name	The Inhibition of NR2B Currents (%)	No.	Name	The Inhibition of NR2B Currents (%)
1	conotoxin-Ac1	51.69 ± 7.62	11	conotoxin-Ac1-E10A	6.54 ± 0.07
2	conotoxin-Ac1-O6P	10.63 ± 1.52	12	conotoxin-Ac1-N11A	14.29 ± 0.93
3	conotoxin-Ac1-N1A	6.43 ± 0.50	13	conotoxin-Ac1-S12A	2.09 ± 0.75
4	conotoxin-Ac1-Y2A	12.54 ± 1.59	14	conotoxin-Ac1-W13A	10.65 ± 0.57
5	conotoxin-Ac1-Y3A	9.90 ±0.54	15	conotoxin-Ac1-W14A	15.72 ± 0.96
6	conotoxin-Ac1-L4A	9.99 ± 0.71	16	conotoxin-Ac1-T15A	13.39 ± 1.29
7	conotoxin-Ac1-Y5A	12.54 ± 0.26	17	conotoxin-Ac1-15 *	3.30 ± 2.51
8	conotoxin-Ac1-O6A	18.98 ± 2.13	18	conotoxin-Ac1-E10γ	4.20 ± 1.61
9	conotoxin-Ac1-R8A	8.12 ± 0.91	19	conotoxin-Ac1-E10γW14γ	5.14 ± 2.68
10	conotoxin-Ac1-O9A	4.27 ± 1.97	20	conotoxin-Ac1-E10γW14γ15 *	7.63 ± 0.50

* C-terminal amidation; γ: γ-carboxyglutamic acid; O: hydroxyproline.

**Table 5:** The overall animal activity of conotoxins conotoxin-Ac1 and conotoxin-Ac1-O6P((Mean ± SD).

Group	Sleep Latency (min)	Sleep Time (min)
control	12.55 ± 1.23	3.27 ± 1.01
conotoxin-Ac1 250µg/kg	3.62 ± 0.54 **	14.81 ± 4.69 **
conotoxin-Ac1 500µg/kg	1.64 ± 0.48 **	36.87 ± 5.82 **
conotoxin-Ac1-O6P 250µg/kg	4.15 ± 2.06 **	12.49 ± 4.28 **
conotoxin-Ac1-O6P 500µg/kg	2.03 ± 1.35 **	21.54 ± 6.72 **

** *p* < 0.01 vs. the control group.

**Table 6:** Analgesic activity of conotoxin-Ac1 and conotoxin-Ac1-O6P, as determined by the hot-plate test ($\bar{x}$ ± s).

No.	Group	Pain Threshold (s)				
		15 min	30 min	60 min	120 min	180 min
	control	15.39 ± 5.03	13.21 ± 4.56	15.14 ± 4.63	12.71 ± 4.64	21.91 ± 4.93
	morpholine 1mg/kg	17.19 ± 2.22	41.80 ± 4.58	50.41 ± 6.59	50.82± 4.35	47.11 ± 4.04
1	conotoxin-Ac1	16.17 ± 2.49	23.16 ± 3.80	34.18 ± 2.35	37.19 ± 7.31	33.51 ± 4.66
2	conotoxin-Ac1-O6P	15.84 ± 1.76	23.19 ± 5.92	38.90 ± 10.09	38.47 ± 6.59	33.44 ± 1.72
3	conotoxin-Ac1-N1A	17.33 ± 3.77	21.34 ± 7.15	34.71 ± 6.91	32.13 ± 9.19	28.26 ± 9.14
4	conotoxin-Ac1-Y2A	16.81 ± 1.56	22.28 ± 3.40	36.77 ± 8.12	33.84 ± 7.51	29.90 ± 7.51
5	conotoxin-Ac1-Y3A	17.56 ± 2.49	23.60 ± 8.28	35.95 ± 7.67	32.14 ± 6.87	28.77 ± 6.32
6	conotoxin-Ac1-L4A	17.70 ± 2.71	23.05 ± 7.12	34.09 ± 6.75	37.28 ± 5.09	34.44 ± 4.78
7	conotoxin-Ac1-Y5A	16.45 ± 4.79	21.88 ± 11.18	31.76 ± 9.33	32.09 ± 6.26	30.40 ± 6.01
8	conotoxin-Ac1-O6A	16.12 ± 2.49	22.39 ± 10.40	31.53 ± 7.59	34.82 ± 8.00	32.03 ± 8.56
9	conotoxin-Ac1-R8A	16.93 ± 3.83	20.24 ± 5.39	31.80 ± 10.83	35.93 ± 7.64	31.89 ± 7.64
10	conotoxin-Ac1-O9A	16.75 ± 3.65	25.45 ± 5.02	29.52 ± 6.69	31.83 ± 8.39	29.70 ± 7.97
11	conotoxin-Ac1-E10A	16.69 ± 3.12	19.84 ± 8.99	28.05 ± 5.57 #	36.62±8.41	31.61 ± 8.41
12	conotoxin-Ac1-N11A	16.67 ± 0.94	25.32 ± 9.81	35.82 ± 7.51	34.24 ± 7.90	32.40 ± 7.90
13	conotoxin-Ac1-S12A	15.73 ± 3.43	25.41 ± 7.77	35.24 ± 6.58	35.20 ± 8.66	31.50±7.95
14	conotoxin-Ac1-W13A	16.48 ± 2.48	20.78 ± 6.92	30.26 ± 10.33	35.83 ± 7.00	32.21 ± 6.44
15	conotoxin-Ac1-W14A	15.96 ± 3.60	21.64 ± 8.98	28.39 ± 5.13	36.42 ± 7.12	31.73 ± 6.40
16	conotoxin-Ac1-T15A	17.78 ± 2.69	19.56 ± 8.59	27.19 ± 7.42 #	30.83 ± 7.13	26.62 ± 6.40
17	conotoxin-Ac1-15 *	16.06 ± 3.31	30.26 ± 7.69	39.59 ± 9.63	41.93 ± 10.04	38.03 ± 9.11
18	conotoxin-Ac1-E10γ	16.35 ± 3.19	26.50 ± 7.79	38.44 ± 8.80	44.33 ± 4.91 ##	36.17 ± 8.28
19	conotoxin-Ac1-E10γW14γ	17.90 ± 2.84	33.94 ± 7.88 #	39.64 ± 5.44 #	47.61 ± 2.26 #	44.46 ± 2.11 ##
20	conotoxin-Ac1- E10γW14γ15 *	15.98 ± 3.55	33.23 ± 3.01 #	51.08 ± 3.52 ##	54.24 ± 3.09 ##	50.55 ± 2.88 ##

* C-terminal amidation; γ: γ-carboxyglutamic acid; O: hydroxyproline; # *p* < 0.05 vs. the control group; ## *p* < 0.01 vs. the control group.

**Table 7:** Summary of conotoxins without cysteine.

Family	Example	Sequence	Target	Ref.
conantokin	conantokin-G	GEγγLQγNQγLIRγKSN *	NMDAR	[18]
	conantokin-T	GEγγYQKMLγNLRγAEVKKNA *	NMDAR	[19]
	conantokin-L	GEγγVAKMAAγLARγDAVN *	NMDAR	[20]
contulakin	contulakin-G	ZSEEGGSNAT†KKPYIL	Neurotensin receptor	[16]
	contulakin-Ca1	MLTKFETKSARVKGLSFHPKRPWVL	unknown	[21]
conorfamide	Conorfamide-Sr1	GPMGWVPVFYRF *	RFamide receptor	[17]
	Conorfamide-Sr2	GPMγDPLγIIRI *	RFamide receptor	[22]
	Conorfamide-Vc1	HSGFLLAWSGPRNRFVRF *	RFamide receptor	[23]
conomap	Conomap-Vt	AFVKGSAQRVAHGY *	unknown	[24]
conophan	conophan gld-V	AOANSVWS	unknown	[25]
	conophan mus-V	SOANS(hVa)WS	unknown	[25]
κ-conotoxin	Mo1659	FHGGSWYRFPWGY *	K ^†^channel	[26]

γ: carboxylated glutamate; *: amidated C-terminus; †: glycosylation; O: hydroxyproline; hVa: gamma-hydroxy-D-valine; F: D-phenylalanine; V: D-hydroxyvaline.

## Supplementary Materials

The following are available online at https://www.mdpi.com/1660-3397/18/3/135/s1, Figure S1: Isolation of crude conotoxins from Conus achatinus, Figure S2: HPLC analysis of fraction 5, Figure S3: The co-elution of synthetic conotoxin-Ac1 and wild conotoxin-Ac1, Figure S4: The co-elution of synthetic conotoxin-Ac1-O6P and wild conotoxin-Ac1-O6P, Table S1: Analgesic activity of conotoxins conotoxin-Ac1 and conotoxin-Ac1-O6P, as determined by the hot-plate test, Table S2: Analgesic activity of conotoxins conotoxin-Ac1 and conotoxin-Ac1-O6P, as determined by the tail-flick test, supplemental MS data: The MS data fugures of synthetic conotoxin-Ac1-O6P and its mutants.
