# Supplementary material for: Structural and Functional Characterization of Conotoxins from Conus achatinus Targeting NMDAR
Source: Mar Drugs. 2020 Feb 26;18(3):135. doi: 10.3390/md18030135 (PMC7143421; doi:10.3390/md18030135)
Supplement: Supplementary file 1 [file marinedrugs-18-00135-s001.zip › Supplemental files/Supplemental Figures.docx]

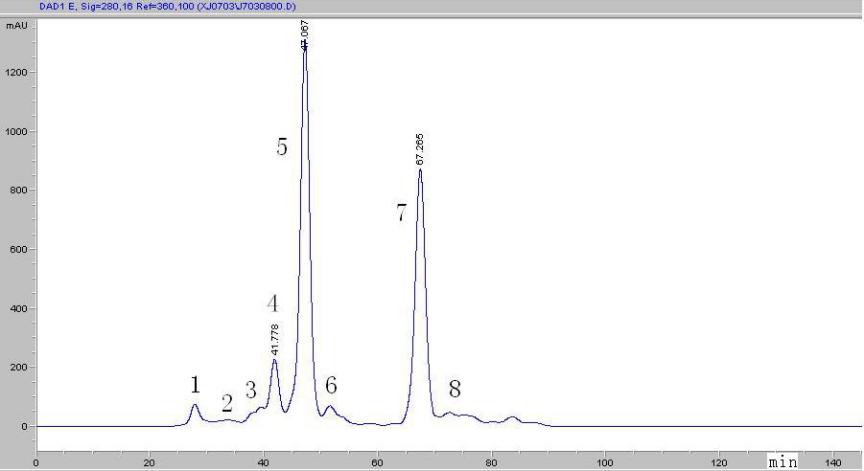


Supplemental Figure 1. Isolation of crude conotoxins from *Conus achatinus*

Separation conditions: mobile phase A: 0.1% TFA; mobile phase B: acetonitrile (0.1% TFA); chromatographic column: Superdex Peptide column; detection wavelength: 254nm / 280nm; flow rate: 0.15ml / min; elution conditions: 30% mobile phase B.


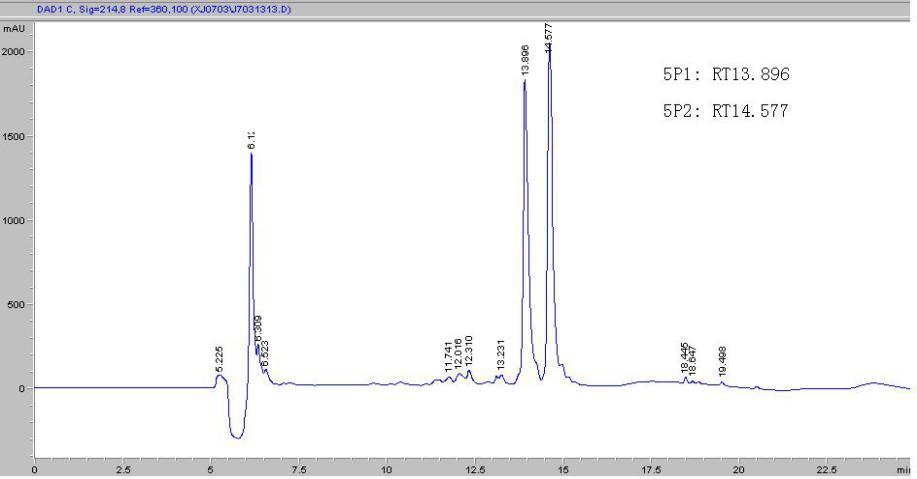


Supplemental Figure 2. HPLC analysis of fraction 5

Separation conditions: mobile phase A: 0.1% TFA; mobile phase B: acetonitrile (0.1% TFA); chromatography column: Zobax semi-preparative column; detection wavelength 214nm; flow rate: 2ml / min; 35-70 / 5min; 70-25 / 5min.


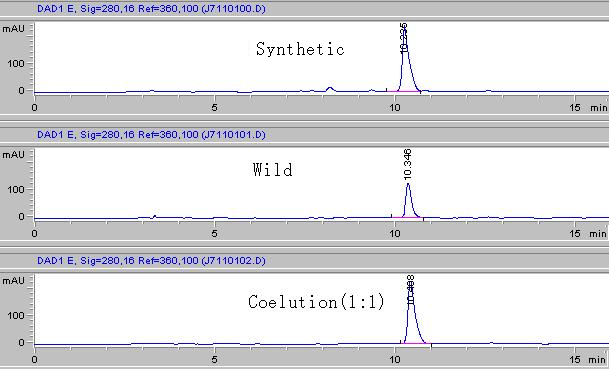


Supplemental Figure 3. The co-elution of synthetic conotoxin-Ac1 and wild conotoxin-Ac1

Separation conditions: mobile phase A: 0.1% TFA; mobile phase B: acetonitrile (0.1% TFA); chromatography column: Vydac analytical column; detection wavelength 214nm; flow rate: 1ml / min; B concentration (%): 25-35 / 10min, 35-70 / 5min, 70-25 / 5min.


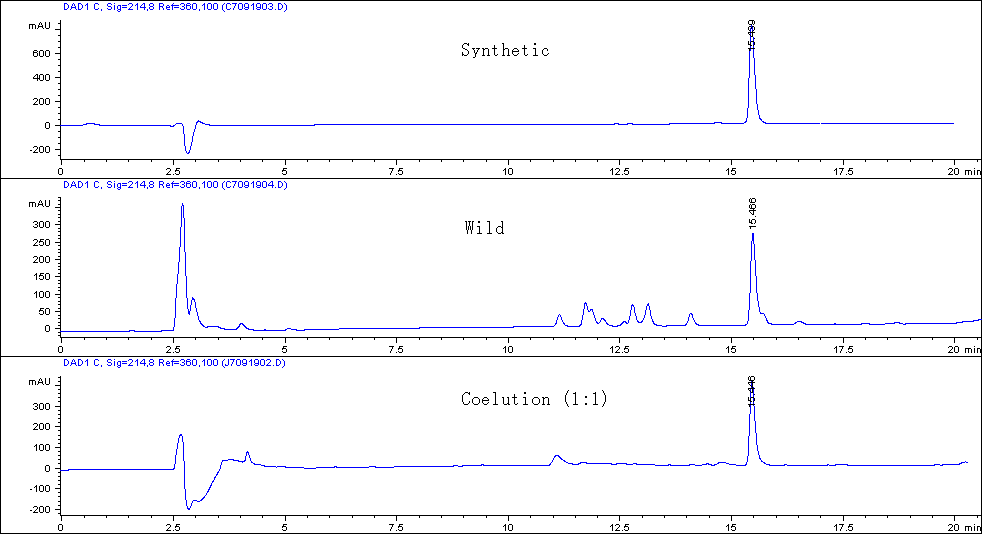


Supplemental Figure 4. The co-elution of synthetic conotoxin-Ac1-O6P and wild conotoxin-Ac1-O6P

Separation conditions: mobile phase A: 0.1% TFA; mobile phase B: acetonitrile (0.1% TFA); chromatography column: Vydac analytical column; detection wavelength 214nm; flow rate: 1ml / min; B concentration (%): 25-35 / 10min, 35-70 / 5min, 70-25 / 5min.
