# Supplementary material for: Structural and Functional Characterization of Conotoxins from Conus achatinus Targeting NMDAR
Source: Mar Drugs. 2020 Feb 26;18(3):135. doi: 10.3390/md18030135 (PMC7143421; doi:10.3390/md18030135)
Supplement: Supplementary file 1 [file marinedrugs-18-00135-s001.zip › Supplemental files/Supplemental MS data.docx]

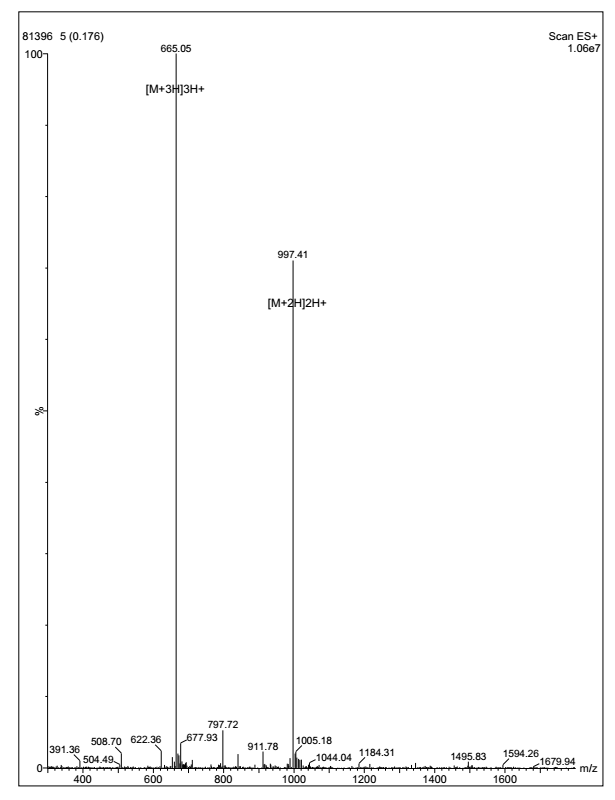

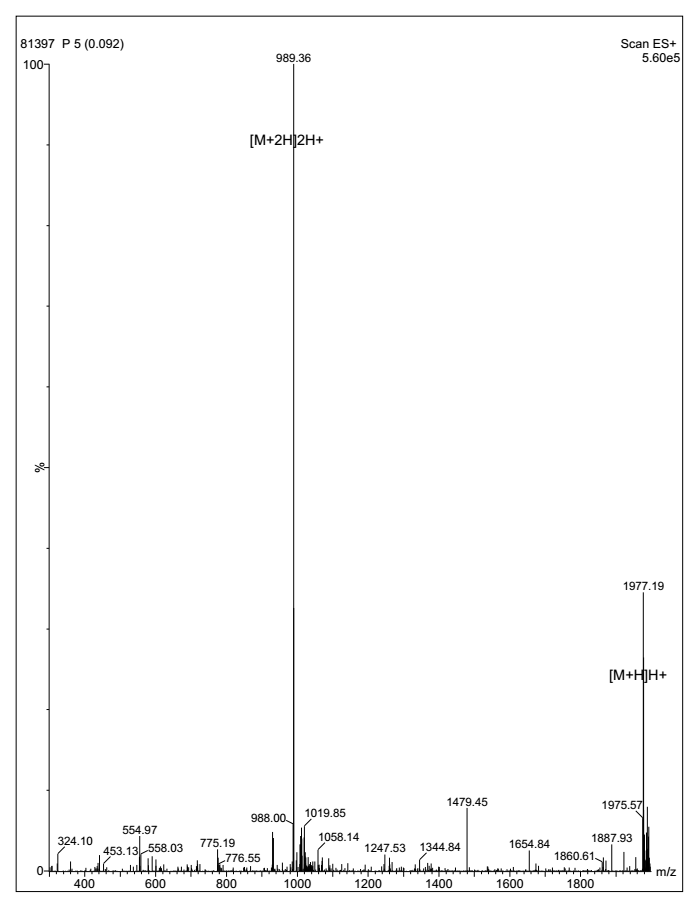


conotoxin-Ac1 conotoxin-Ac1-O6P


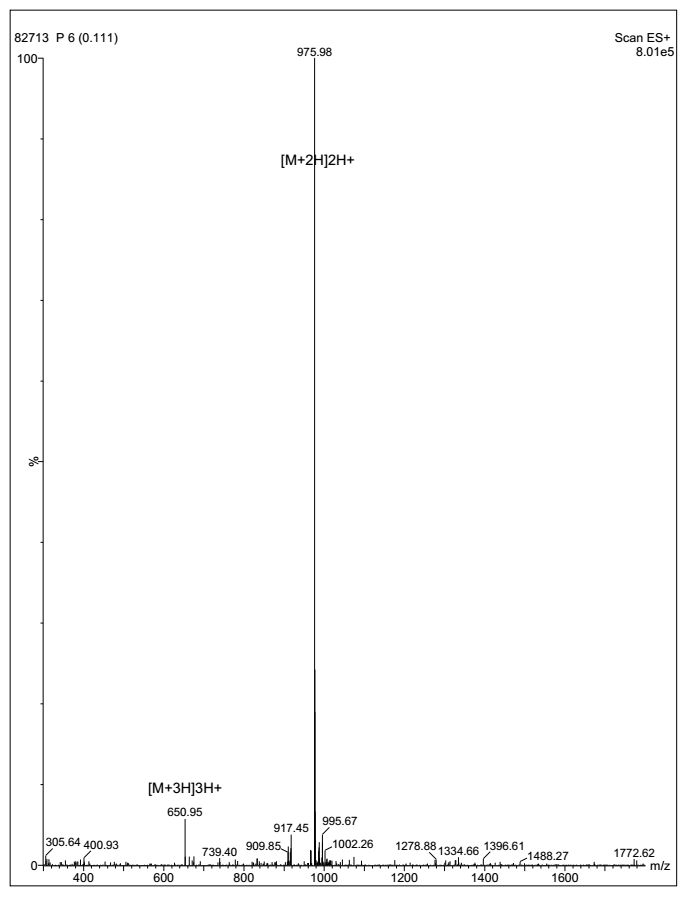

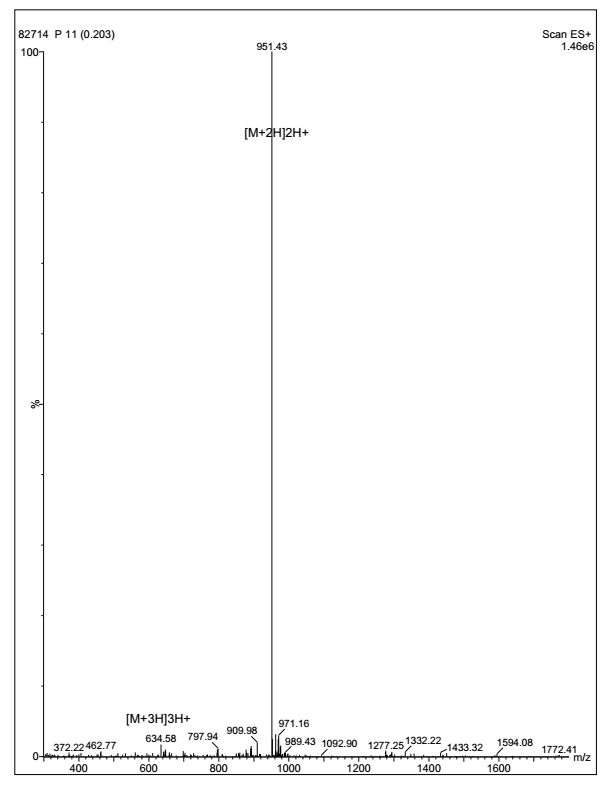


conotoxin-Ac1-N1A conotoxin-Ac1-Y2A


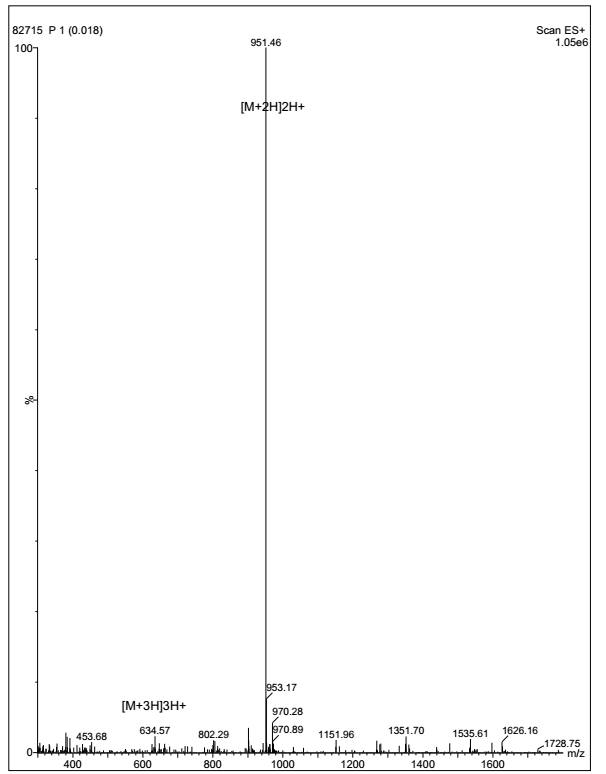

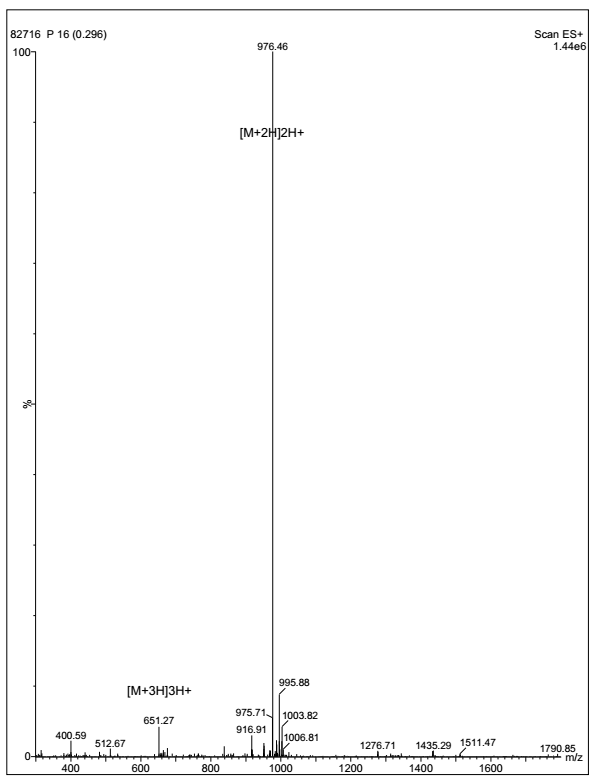


conotoxin-Ac1-Y3A conotoxin-Ac1-L4A


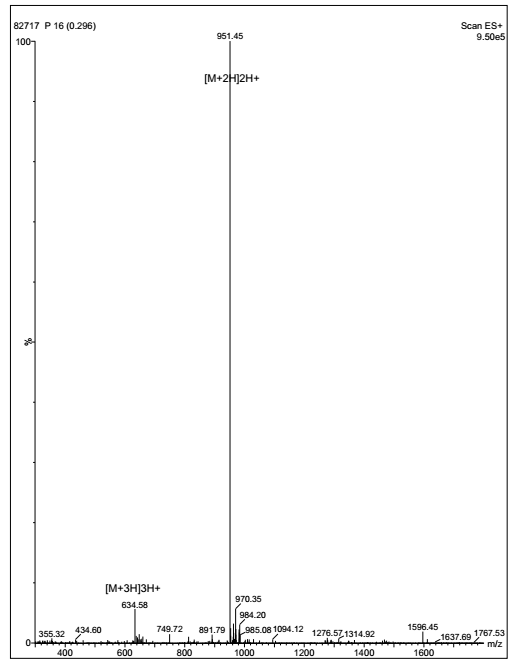

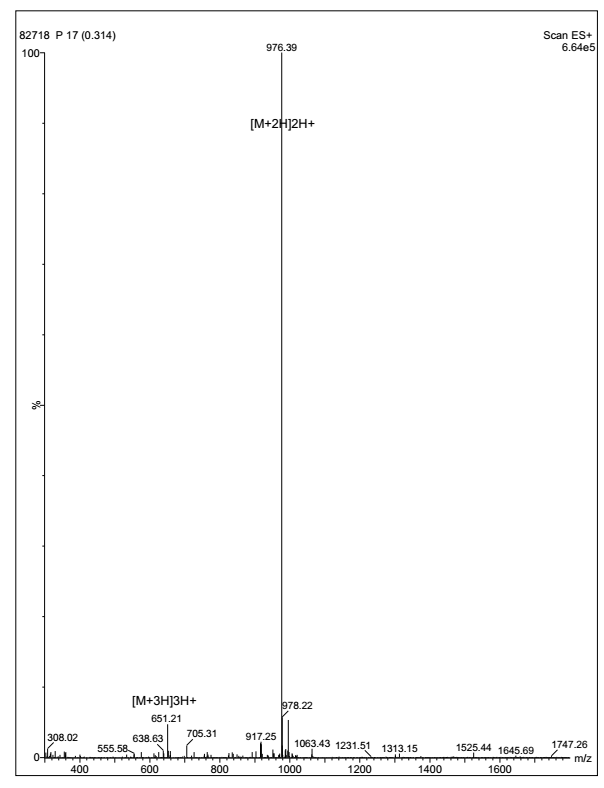


conotoxin-Ac1-Y5A conotoxin-Ac1-O6A


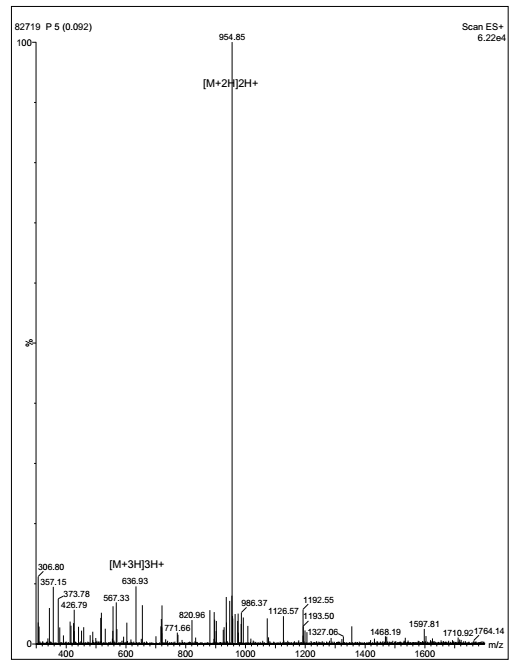

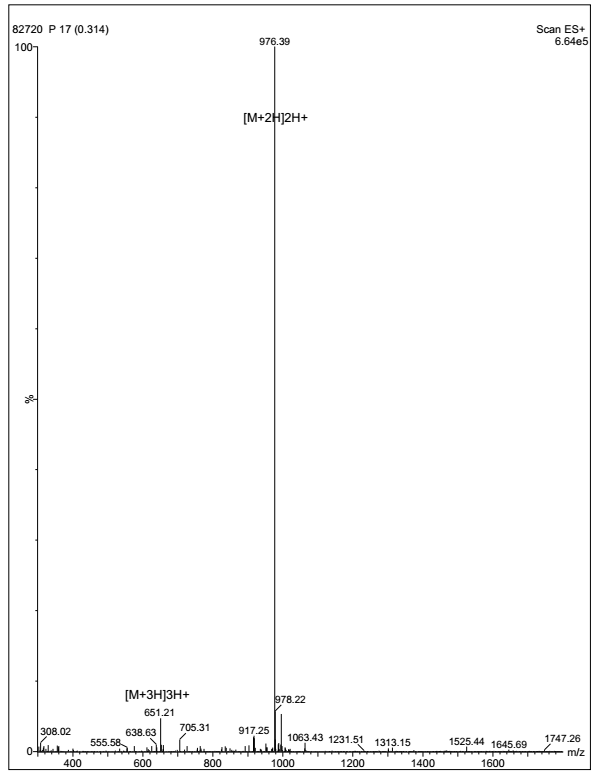


conotoxin-Ac1-R8A conotoxin-Ac1-O9A


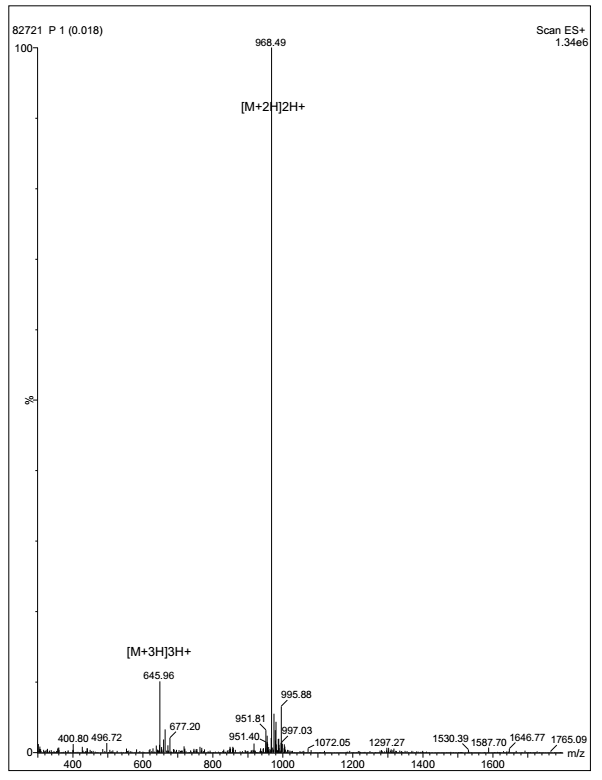

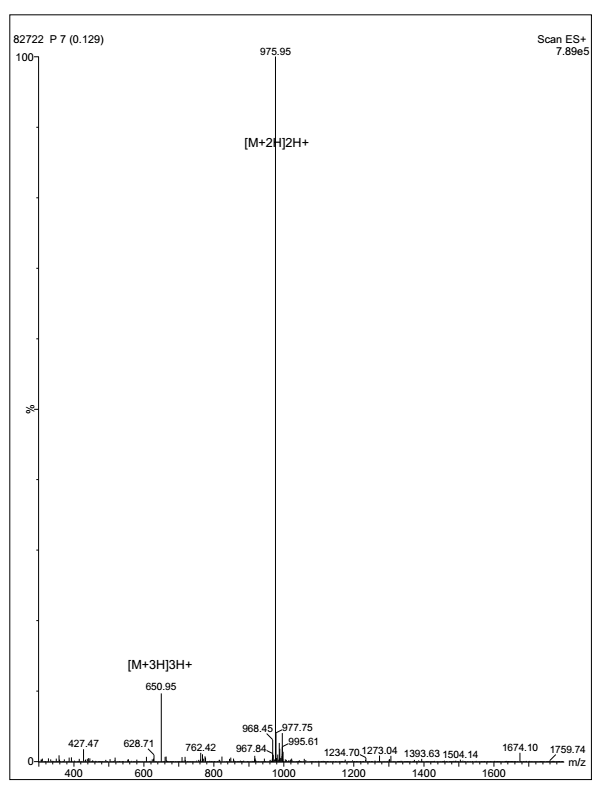


conotoxin-Ac1-E10A conotoxin-Ac1-N11A


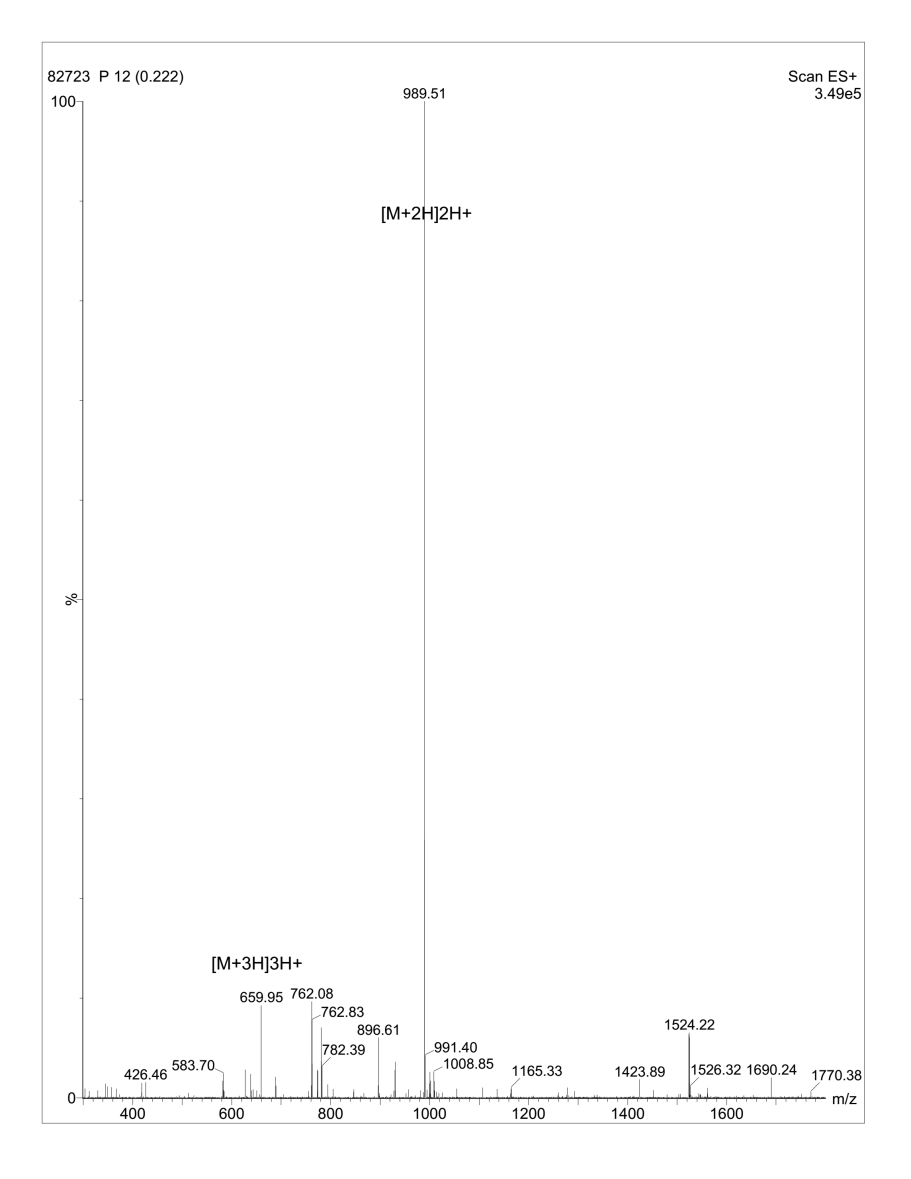

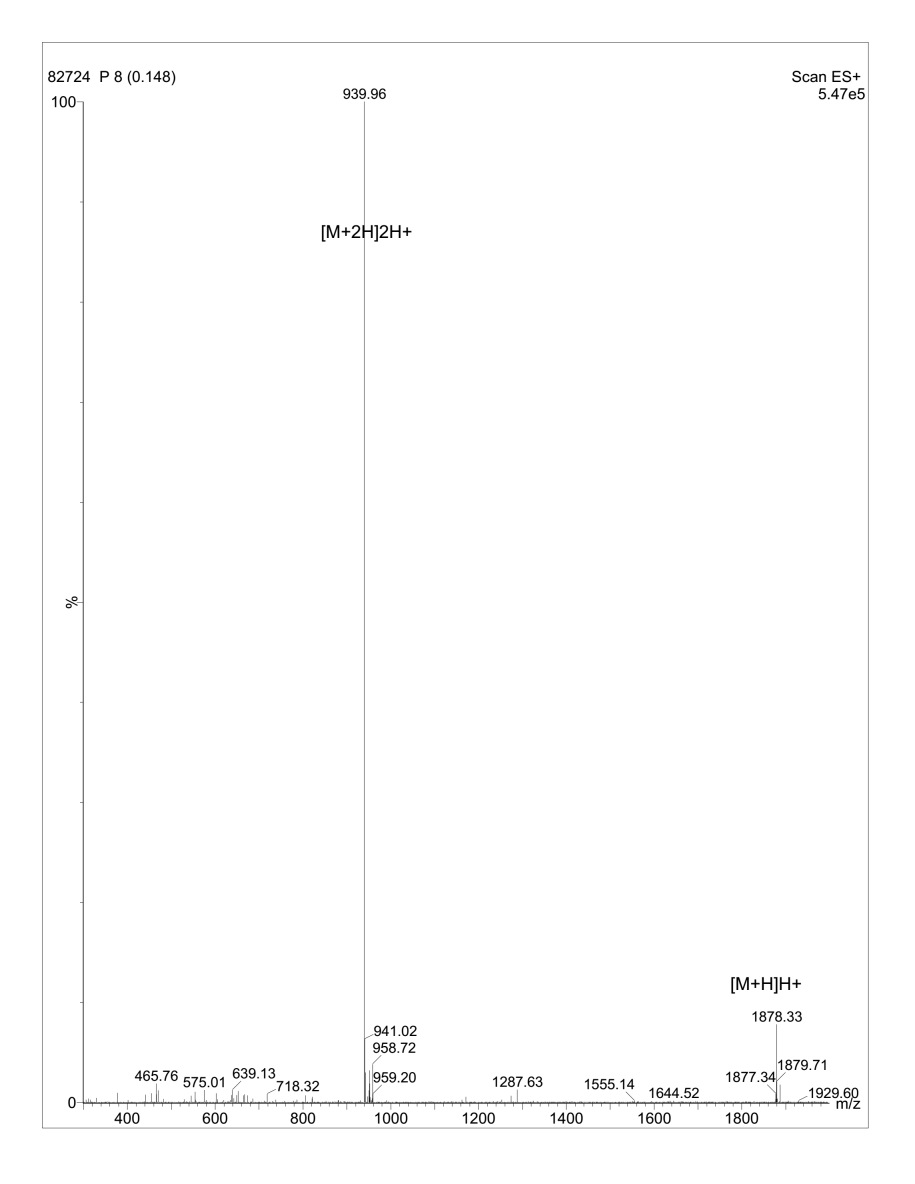


conotoxin-Ac1-S12A conotoxin-Ac1-W13A


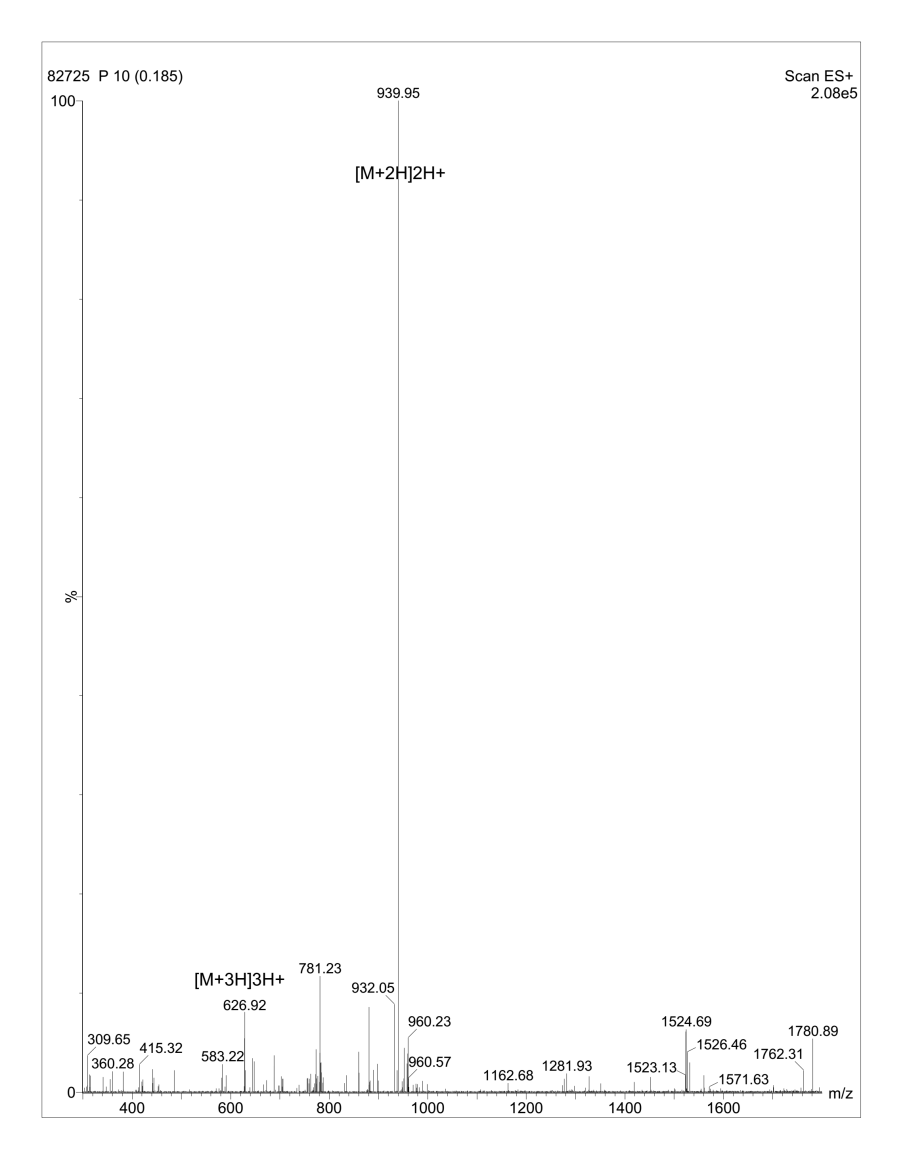

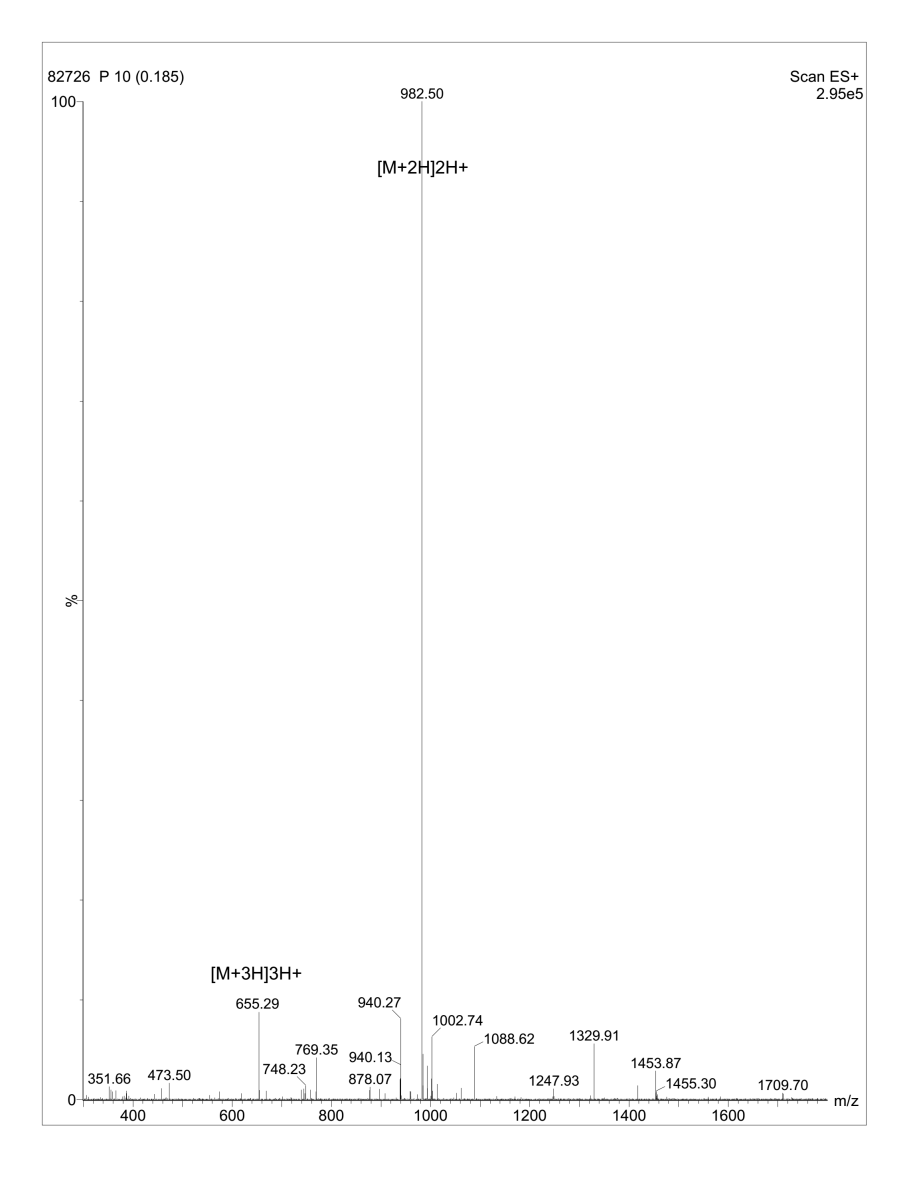


conotoxin-Ac1-W14A conotoxin-Ac1-T15A


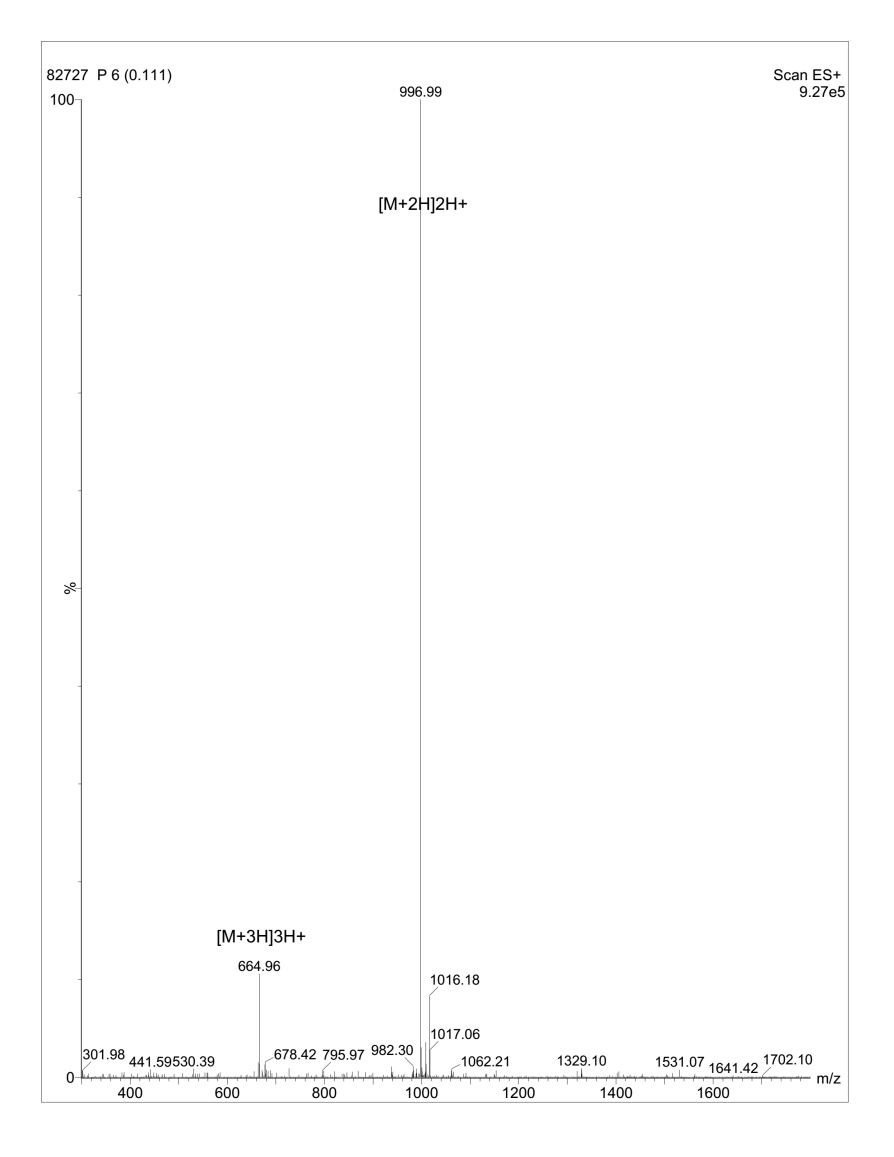

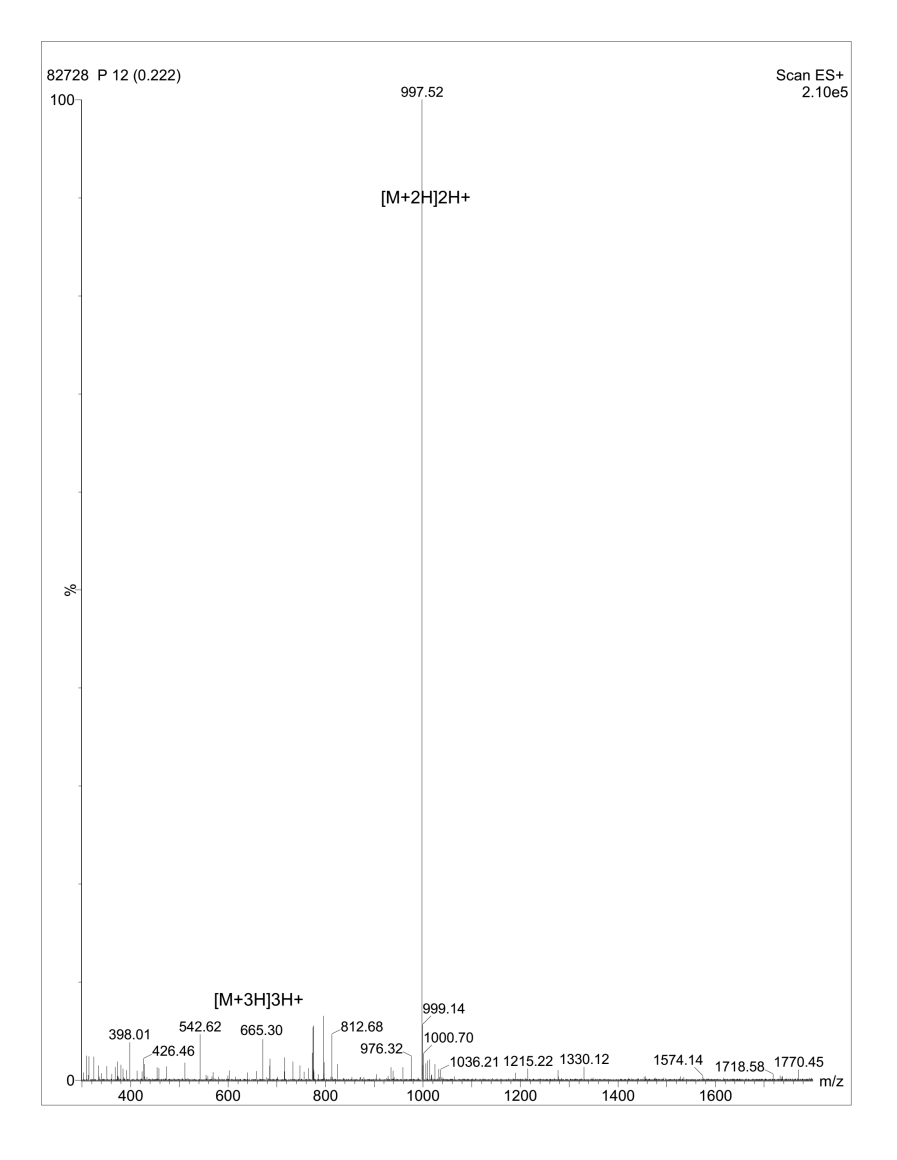


conotoxin-Ac1-15* conotoxin-Ac1-E10γ


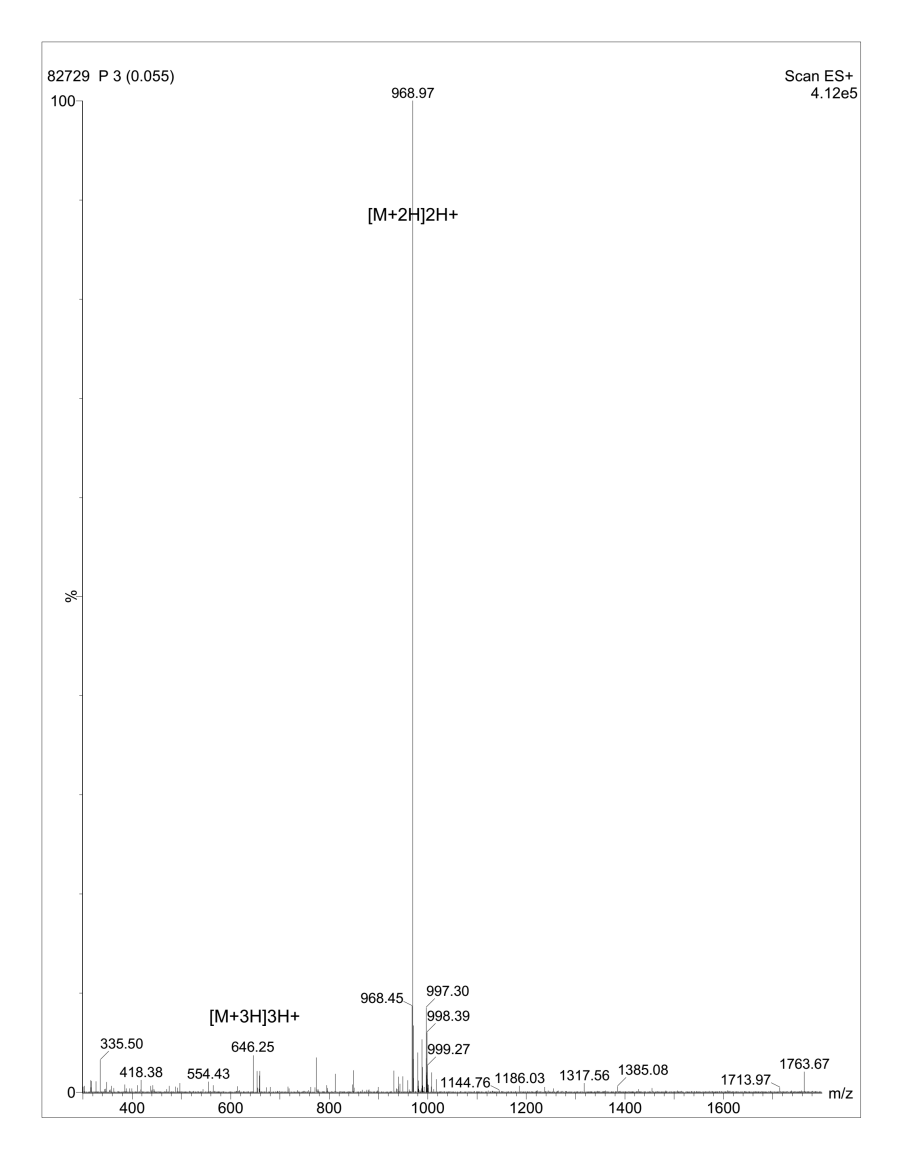

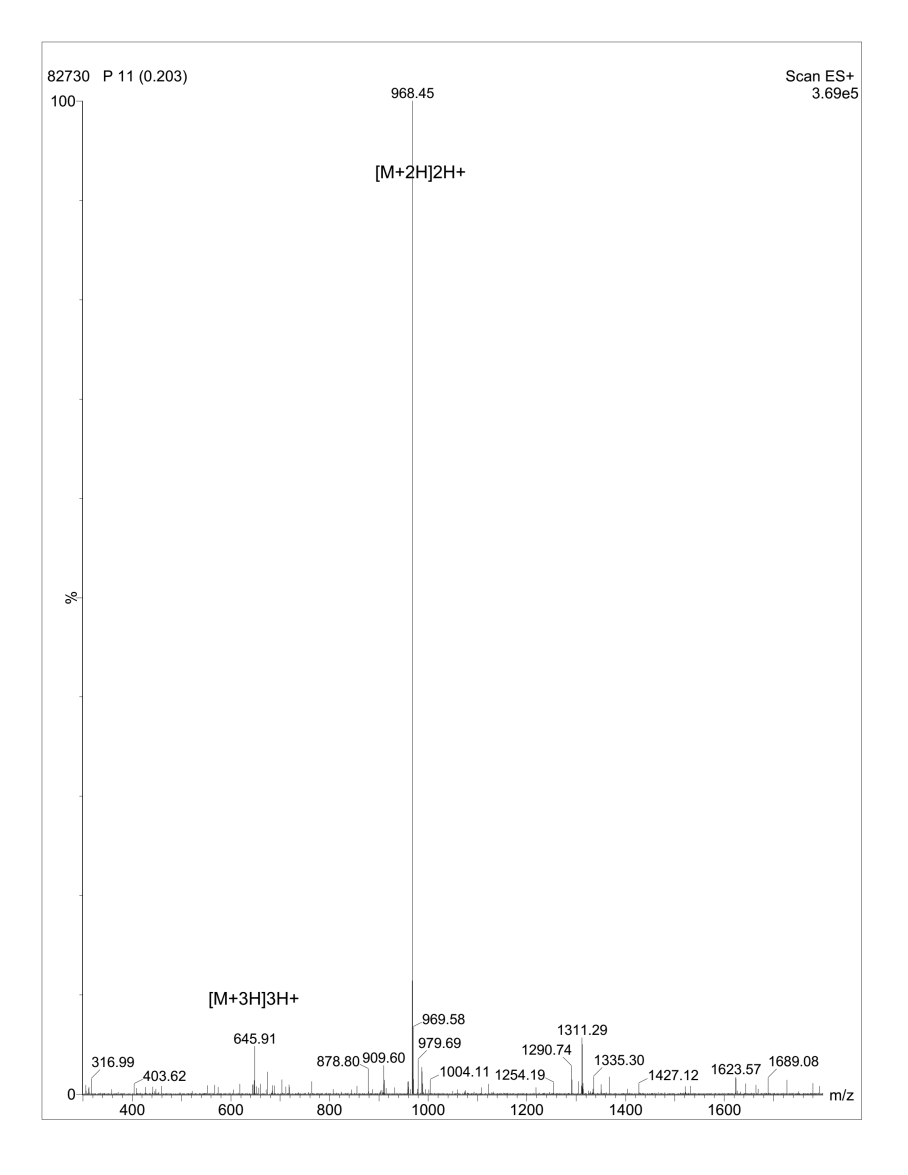


conotoxin-Ac1-E10γW14γ conotoxin-Ac1-E10γW14γ15*
