# Supplementary material for: Structural and Functional Characterization of Conotoxins from Conus achatinus Targeting NMDAR
Source: Mar Drugs. 2020 Feb 26;18(3):135. doi: 10.3390/md18030135 (PMC7143421; doi:10.3390/md18030135)
Supplement: Supplementary file 1 [file marinedrugs-18-00135-s001.zip › Supplemental files/Supplemental table.docx]

Supplemental Table 1. Analgesic activity of conotoxins 5P1 and 5P2, as determined by the hot-plate test, in mice ( ± s).


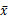

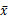


| group | pain threshold (s) | | | | |
| --- | --- | --- | --- | --- | --- |
|  | 15min | 30min | 60min | 120min | 180min |
| control | 15.39±5.03 | 15.09±7.35 | 16.81±7.94 | 17.35±5.37 | 21.91±4.93 |
| morpholine 1mg/kg | 17.19±2.22 | 41.80±4.58** | 50.41±6.59** | 50.82±4.35** | 47.11±4.04** |
| 5P1 10μg/kg | 16.17±2.49 | 23.16±3.80** | 34.18±2.35** | 37.19±7.31** | 33.51±4.66** |
| 5P1 20μg/kg | 16.22±1.87 | 22.88±5.93** | 37.29±2.85** | 39.73±10.84** | 38.97±8.06** |
| 5P1 40μg/kg | 16.77±2.86 | 28.36±4.73** | 38.64±1.69** | 44.87±6.09** | 43.77±6.03** |
| 5P2 10μg/kg | 16.08±2.71 | 23.19±5.92** | 38.90±10.09** | 38.47±6.59** | 32.94±3.19** |
| 5P2 20μg/kg | 17.78±2.38 | 22.98±5.32** | 40.82±5.59** | 43.35±5.92** | 39.24±7.81** |
| 5P2 40μg/kg | 18.85±3.01 | 29.39±4.35** | 45.06±5.62** | 48.90±6.55** | 44.71±6.37** |

* p<0.05 vs the control group, ** p<0.01 vs the control group

Supplemental Table 2. Analgesic activity of conotoxins 5P1 and 5P2, as determined by the tail-flick test ( ± s).


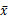

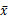


| group | pain threshold (s) | | | | |
| --- | --- | --- | --- | --- | --- |
|  | 15min | 30min | 60min | 120min | 180min |
| control | 1.13±0.36 | 1.37±0.40 | 1.19±0.46 | 1.23±0.38 | 1.33±0.44 |
| 5P1 10μg/kg | 1.53±0.27** | 1.93±0.27** | 2.46±0.39** | 2.79±0.53** | 2.77±0.59** |
| 5P1 20μg/kg | 1.80±0.27** | 2.22±0.29** | 3.37±0.58** | 3.34±0.35** | 3.10±0.42** |
| 5P1 40μg/kg | 1.91±0.20** | 2.32±0.17** | 4.08±0.53** | 3.87±0.33** | 3.59±0.89** |
| 5P2 10μg/kg | 1.77±0.18** | 2.17±0.18** | 2.45±0.65** | 2.51±0.75** | 2.27±0.46** |
| 5P2 20μg/kg | 1.81±0.24** | 2.26±0.23** | 3.12±0.88** | 3.06±0.87** | 2.92±0.45** |
| 5P2 40μg/kg | 2.00±0.20** | 2.45±0.18** | 4.05±0.28** | 3.90±0.76** | 3.57±0.48** |

* p<0.05 vs the control group, ** p<0.01 vs the control group
